# Proceedings of the Second Curing Coma Campaign NIH Symposium: Challenging the Future of Research for Coma and Disorders of Consciousness

**DOI:** 10.1007/s12028-022-01505-3

**Published:** 2022-05-10

**Authors:** Shraddha Mainali, Venkatesh Aiyagari, Sheila Alexander, Yelena Bodien, Varina Boerwinkle, Melanie Boly, Emery Brown, Jeremy Brown, Jan Claassen, Brian L. Edlow, Ericka L. Fink, Joseph J. Fins, Brandon Foreman, Jennifer Frontera, Romergryko G. Geocadin, Joseph Giacino, Emily J. Gilmore, Olivia Gosseries, Flora Hammond, Raimund Helbok, J. Claude Hemphill, Karen Hirsch, Keri Kim, Steven Laureys, Ariane Lewis, Geoffrey Ling, Sarah L. Livesay, Victoria McCredie, Molly McNett, David Menon, Erika Molteni, DaiWai Olson, Kristine O’Phelan, Soojin Park, Len Polizzotto, Jose Javier Provencio, Louis Puybasset, Chethan P. Venkatasubba Rao, Courtney Robertson, Benjamin Rohaut, Michael Rubin, Tarek Sharshar, Lori Shutter, Gisele Sampaio Silva, Wade Smith, Robert D. Stevens, Aurore Thibaut, Paul Vespa, Amy K. Wagner, Wendy C. Ziai, Elizabeth Zink, Jose I Suarez, the Curing Coma Campaign collaborators

**Affiliations:** 1 Department of Neurology, Virginia Commonwealth University School of Medicine, Richmond, VA USA; 2 Neurological Surgery and Neurology, University of Texas Southwestern Medical Center, Dallas, TX USA; 3 School of Nursing, University of Pittsburgh, Pittsburgh, PA USA; 4 Department of Physical Medicine and Rehabilitation, Spaulding Rehabilitation Hospital and Harvard Medical School, Charlestown, MA USA; 5 Division of Neurology, Barrow Neurological Institute at Phoenix Children’s Hospital, Phoenix, AZ USA; 6 Departments of Neurology and Psychiatry, Wisconsin Institute for Sleep and Consciousness, University of Wisconsin, Madison, WI USA; 7 Department of Anesthesia, Critical Care and Pain Medicine, Massachusetts General Hospital and Harvard Medical School, Boston, MA USA; 8 Office of Emergency Care Research, Division of Clinical Research, National Institute of Neurological Disorders and Stroke, Bethesda, MD USA; 9 Department of Neurology, Columbia University Medical Center, New York Presbyterian Hospital, New York, NY USA; 10 Department of Neurology, Center for Neurotechnology and Neurorecovery, Massachusetts General Hospital, Boston, MA USA; 11 Department of Critical Care Medicine, UPMC Children’s Hospital of Pittsburgh, University of Pittsburgh Medical Center, Pittsburgh, PA USA; 12 Division of Medical Ethics, Weill Cornell Medical College, New York, NY USA; 13 Division of Neurocritical Care, Department of Neurology and Rehabilitation Medicine, University of Cincinnati, Cincinnati, OH USA; 14 Department of Neurology, New York University School of Medicine, New York, NY USA; 15 Division of Neurosciences Critical Care, Departments of Anesthesiology and Critical Care Medicine, Neurology, and Neurosurgery, The Johns Hopkins University School of Medicine, Baltimore, MD USA; 16 Harvard Medical School, Spaulding Rehabilitation Hospital, Boston, MA USA; 17 Comprehensive Epilepsy Center, Department of Neurology, Yale University, New Haven, CT USA; 18 Coma Science Group, GIGA Consciousness, University of Liege, Liege, Belgium; 19 Indiana University Department of Physical Medicine and Rehabilitation, University of Indiana School of Medicine, Indianapolis, IN USA; 20 Department of Neurology, Medical University of Innsbruck, Innsbruck, Austria; 21 Department of Neurology, University of California, San Francisco, CA USA; 22 Division of Neurocritical Care, Department of Neurology, Stanford University, Stanford, CA USA; 23 College of Pharmacy, University of Illinois, Chicago, IL USA; 24 Coma Science Group, Cyclotron Research Center, University of Liege, Liege, Belgium; 25 Department of Neurology and Neurosurgery, New York University Langone Health, New York, NY USA; 26 Department of Adult Health and Gerontological Nursing, College of Nursing, Rush University, Chicago, IL USA; 27 Interdepartmental Division of Critical Care, Department of Respirology, University of Toronto, Toronto, ON Canada; 28 College of Nursing, Ohio State University, Columbus, OH USA; 29 Division of Anaesthesia, University of Cambridge, Cambridge, UK; 30 School of Biomedical Engineering and Imaging Sciences, King’s College London, London, UK; 31 Neuroscience Intensive Care Unit, O’Donnell Brain Institute, University of Texas Southwestern Medical Center, Dallas, TX USA; 32 Department of Neurology, Miller School of Medicine, University of Miami, Miami, FL USA; 33 Department of Neurology and Neurocritical Care, Columbia University, New York, NY USA; 34 Department of Biomedical Engineering, Worcester Polytechnic Institute, Worcester, MA USA; 35 Department of Neurology and Neuroscience, University of Virginia, Charlottesville, VA USA; 36 Department of Neuroradiology, University of Paris VI, Pierre et Marie Curie, Pitié-Salpêtrière Hospital, Paris, France; 37 Division of Vascular Neurology and Neurocritical Care, CHI St. Luke’s Health–Baylor St. Luke’s Medical Center, Baylor College of Medicine, Houston, TX USA; 38 Departments of Anesthesiology and Critical Care Medicine, and Pediatrics, Johns Hopkins Children’s Center, The Johns Hopkins University School of Medcine, Baltimore, MD USA; 39 Neuroscience Intensive Care Unit, Department of Neurology, Sorbonne University, Assistance Publique–Hôpitaux de Paris, Pitié-Salpêtrière Hospital, Paris, France; 40 Department of Intensive Care, Paris Descartes University, Paris, France; 41 University of Pittsburgh, Pittsburgh, PA USA; 42 Hospital Israelita Albert Einstein, Academic Research Organization and Department of Neurology and Neurosurgery, Universidade Federal de São Paulo, São Paulo, Brazil; 43 Ronald Reagan UCLA Medical Center, UCLA Santa Monica Medical Center, Santa Monica, CA USA; 44 Department of Physical Medicine and Rehabilitation, School of Medicine, University of Pittsburgh, Pittsburgh, PA USA; 45 Department of Neuroscience Nursing, The Johns Hopkins Hospital, The Johns Hopkins University, Baltimore, MD USA; 46 Athinoula A. Martinos Center for Biomedical Imaging, Massachusetts General Hospital, Charlestown, MA USA; 47 Yale Law School, New Haven, CT USA; 48 Centre du Cerveau, University Hospital of Liege, Liege, Belgium; 49 Department of Neurology, Centre Hospitalier Universitaire Sart Tilman, University of Liege, Liege, Belgium

**Keywords:** Disorders of consciousness, Coma, Curing Coma Campaign, National Institute of Health, Proceedings

## Abstract

This proceedings article presents actionable research targets on the basis of the presentations and discussions at the 2nd Curing Coma National Institutes of Health (NIH) symposium held from May 3 to May 5, 2021. Here, we summarize the background, research priorities, panel discussions, and deliverables discussed during the symposium across six major domains related to disorders of consciousness. The six domains include (1) Biology of Coma, (2) Coma Database, (3) Neuroprognostication, (4) Care of Comatose Patients, (5) Early Clinical Trials, and (6) Long-term Recovery. Following the 1st Curing Coma NIH virtual symposium held on September 9 to September 10, 2020, six workgroups, each consisting of field experts in respective domains, were formed and tasked with identifying gaps and developing key priorities and deliverables to advance the mission of the Curing Coma Campaign. The highly interactive and inspiring presentations and panel discussions during the 3-day virtual NIH symposium identified several action items for the Curing Coma Campaign mission, which we summarize in this article.

**Supplementary Information:**

The online version contains supplementary material available at 10.1007/s12028-022-01505-3.

## Introduction

The word “coma” is derived from the Greek word “Koma,” meaning deep sleep. The term was initially used in Hippocratic medicine and then in the second century by Galen before reappearing in the seventeenth century to describe the state of “lack of consciousness” [1]. As provided by Plum and Posner [2], the current definition is a state of unresponsiveness in which the patient lies with eyes closed and cannot be aroused to respond appropriately to stimuli even with vigorous stimulation. The term coma encompasses a spectrum of severity of illness due to a variety of modes and mechanisms of injury but excludes brain death and is distinct from locked-in-state in which consciousness is preserved. In this article, the term “disorders of consciousness” (DoC) is used to broadly capture coma and consciousness-related disorders.

In 1972, the term “persistent vegetative state” was used to describe the state of apparent wakefulness without awareness where responsiveness is limited to primitive postural and reflex movements of the limbs [3]. Other forms of DoC between coma and normal consciousness were vaguely defined and described using variable terminologies such as stupor, lethargy, and clouding of consciousness among others. In 1974, Teasdale and Jennett [4] described the now famous Glasgow Coma Scale (GCS) to address some of the ambiguities surrounding the spectrum of DoC. In 2002, Giacino et al. [5] introduced the concept of “minimally conscious state” (MCS), defined as the condition of severely altered consciousness in which minimal but definite behavioral evidence of self or environmental awareness is demonstrated. Subsequently, the Coma Recovery Scale, Revised (CRS-R) was introduced in 2004 to describe the nuances within the spectrum of DoC and included assessment of the auditory, visual, motor, verbal, communication, and arousal functions [6].

From the primitive understanding of DoC in the seventeenth century CE with the practice of “bloodletting” therapy for stroke, to the modern medicine involving multimodal and multidisciplinary care approach, our understanding of the biology of coma has improved significantly with improved diagnostic and monitoring tools. In 2006, Owen et al. [7] published a breakthrough study using functional magnetic resonance imaging (fMRI) in which evidence of awareness was noted in a patient clinically determined to be in vegetative state. The study was later verified in 2010 by Monti et al. [8] by evaluating 54 patients with DoC, in which the investigators found evidence of awareness in 5 of those patients. Following these discoveries, scientists across the world started various active, passive, and resting state analyses using MRI, electroencephalography (EEG) and fluorodeoxyglucose positron emission tomography techniques to seek evidence of awareness in patients with DoC [9–12]. The term “cognitive motor dissociation” was subsequently introduced in 2015 to describe the phenomenon of covert awareness in patients who are clinically unresponsive [13]. Additional terms such as “functional locked-in” or “MCS” [12, 14] have also been proposed when there is a dissociation between behavior and brain imaging results. A meta-analysis including more than 1000 patients in 2016 suggested that about 15% of patients in apparent vegetative/unresponsive state, in fact, have preserved consciousness [15]. More recently, Claassen et al. [16] again verified the findings of covert awareness (brain activation in response to spoken commands, detected by EEG) in about 15% of patients with vegetative/unresponsive state and noted that covert awareness is associated with improved outcomes. In particular, investigators noted that at 12 months, 44% of patients with notable brain activation on EEG (to verbal commands) had Glasglow Outcome Scale-Extended of 4 or higher (i.e., ability to function independently for 8 or more hours) compared with only 14% of patients without brain activation on EEG (odds ratio, 4.6; 95% confidence interval, 1.2 to 17.1). A new classification of DoC has also recently been proposed by Naccache et al. [17] that combines behavioral evidence with functional brain imaging data to assess residual consciousness. Overall, the field of coma science has seen substantial momentum over the past two decades, which makes this an opportune time to harness global scientific efforts through a centralized platform to improve the lives of patients suffering from DoC.

## The Patient’s Perspective

Patient and family perspectives are integral to curing coma [18]. The symposium attendees heard a patient’s perspective from a cardiac arrest (CA) survivor who shared his remarkable and inspiring journey of recovery and resilience, despite only a 10% expected chance of survival after suffering an out-of-hospital CA. He spoke about the importance of meeting the patients beyond their acute medical needs. He emphasized that medical care becomes most rewarding when compassionate care is provided while integrating patients and families into decision-making. He also highlighted the importance of care provider role across the care spectrum, starting with bystanders who performed cardiopulmonary resuscitation (CPR) to the emergency department and intensive care unit (ICU) teams who did not give up on him despite his prolonged coma and the rehabilitation team that helped him achieve his independence. Recognizing that his survival may not have been possible without the help of bystanders who performed CPR, he founded “Make BLS Basic,” an organization focused on training the general public in basic life support including hands-only CPR training through community events and educational forums [19].

## Neurocritical Care Society in the Forefront of Curing Coma

Despite the advances in mechanistic understandings, improved definitions, new tools, and revised clinical scales over the past five decades, management strategies and patient outcomes of comatose patients have not significantly improved. With the availability of diagnostic tools such as MRI, fluorodeoxyglucose positron emission tomography, and the EEG—along with some promising pharmacologic interventions to aid in coma recovery—the scientific community is more prepared than ever to undertake this mission to advance and improve the care of coma patients. As specialists with rigorous clinical training and dedicated careers in managing critically ill patients with coma and DoC, the neurocritical care community is optimally positioned to lead this mission. In 2016, the leaders of the Neurocritical Care Research Central (NCRC) and the Neurocritical Care Research Network of Neurocritical Care Society (NCS) identified important gaps in the organizational research portfolio of coma and DOC and envisioned an approach to bridge this gap constructively. This effort led to the Blue Ocean Strategy Meeting in 2018 (Boca Raton, Florida), where a panel of multidisciplinary experts collectively agreed on the need for a collaborative approach to “cure coma.” Recognizing the need for a centralized platform to advance coma science, the Curing Coma Campaign (CCC) started with idea generation, expert consensus, and strategic planning. The CCC executive committee and the Scientific Advisory Council have worked diligently to build the infrastructure, identify, and recruit field experts and define important knowledge gaps to embark upon this ardent journey of improving care standards for patients with DoC [20]. With this goal, the CCC was formally launched in 2019 at the NCS Annual Meeting in Vancouver, BC, Canada.

The National Institute of Neurological Disorders and Stroke (NINDS) initially collaborated with the CCC to organize a part I, 2-day virtual symposium in September 2020 to facilitate a broad discussion between various stakeholders, including CCC leadership, National Institutes of Health (NIH) representative, experts in coma science, industry partners, and patient advocates. The summary of the proceedings was published in the special CCC issue [21]. Following the discussions of the first NIH symposium, six major workgroups (WGs) were created to investigate gaps and develop research priorities across six domains: (1) Biology of Coma, (2) Coma Database, (3) Neuroprognostication, (4) Care of Comatose Patients, (5) Early Clinical Trials, and (6) Long-term Recovery. A part II, 3-day virtual symposium took place on May 3–5, 2021. The symposium was well attended with invited participation from 154 field experts and CCC stakeholders from diverse clinical and research backgrounds on the 1st day, 127 participants on the 2nd day, and on the final day of conference briefing, a smaller group of 68 participants were invited to share their viewpoints. The symposium agenda is included in the supplementary material (Appendix 1). The list of participants is also included in the supplementary material (Appendix 2). This proceedings article summarizes the presentations and discussions and presents action items for each of the six domains that are critical to the mission of the CCC.

## Biology of Coma and Consciousness

### Background

The CCC launched the biology of coma WG tasked with determining the state of the science in DoC. The WG was divided into four subgroups whose remits were to (1) account for DoC heterogeneity to refine endotypes, (2) characterize DoC mechanisms by multiple spatial–temporal scales, (3) move Curing Coma science toward individualized prognostic tools, and (4) develop systemic frameworks to optimize therapeutic trials. Details related to recommendations from each of the subgroups were published separately [22–25].

Understanding DoC involves discerning its critical components that include key anatomical sites, systems and connections, characteristics of transmitted information, the neurochemical substrates and the biology that drives human consciousness. The current understanding of DoC along these five categories were discussed:

1. There are specific sites critical for consciousness, but the absence of activation of these sites on functional imaging of patients with DoC (e.g., under propofol anesthesia [28]) does not reliably correlate with absent function [26, 27]. Rostral brainstem, midline thalamic nuclei, and anterior insula have emerged as possible key sites critical for consciousness [28–30]. Yet, it is unclear whether all of these sites, in isolation, are both necessary and sufficient.
2. There are systems and connections that subserve consciousness. However, consciousness is likely a function of individual traits (strength of baseline internodal connectivity) and disease states (i.e., severity of injury or degree of sedation [31]), which should be considered while assessing perception and motor responses in patients with MCS and cognitive motor dissociation (CMD). Some important pathways relevant in DoC include the midline thalamocortical connectivity, the default mode network (DMN) (the neuronal network of cortical regions that are active at rest), brainstem arousal pathways, and the basal mesocircuit [32–34].
3. There are important characteristics of information transmitted by the systems and connections that subserve consciousness. There is a complex assignment of neural resources for task performance. Studies of neural correlates of consciousness demonstrate anticorrelations (negative correlations) between DMN and task-positive network in DoC. These anticorrelations serve as biomarkers of efficient resource allocation and indicate that the content of information processed is relevant for transmission [35]. Consciousness demonstrates the global neuronal integration with the ability to rapidly swap states and occupy functional states or brain configurations outside the constraints of anatomical connectivity [36].
4. What are the neurochemical substrates that modulate this process?
  1. Brainstem arousal systems are important, with diverse roles and neurochemical signatures matched to function [37].
  2. Cortical interneurons are targets of neurochemical modulation [38], and the understanding of this interaction may provide therapeutic opportunities [39].
5. What do we know about biology that drives human consciousness? We know that there are genetic accompaniments to brain structures and systems associated with human evolution [40].

### Research Priorities for Biology of Coma

The biology of coma WG specifically identified five research priorities to accurately understand, diagnose, classify, prognosticate, and develop targeted therapies for patients with DoC.

1. A framework for differentiating clinical subtypes of DoC is currently lacking. The WG proposed clinical differentiation of DoC based on the presence or absence of consciousness, environmental connectedness (awareness of the external environment that influences the content of one’s consciousness), and responsiveness (C-EC-R) [22]*.* Identification of mechanisms involved in each phase of DoC across the C-EC-R spectrum would facilitate the development of targeted therapies specific to that phase.
2. As different regions of the brain are intricately connected in a complex fashion, focal structural changes can lead to distant or even global functional impact (diaschisis). There is a need to identify links between structural brain damage and related functional implications. The development of a comprehensive brain map using multimodal tools such as structural and functional neuroimaging, neurophysiological techniques, and whole-brain computational models is required to understand mechanisms of DoC on a macroscale. Various approaches such as the information theory, graph theory, and the dynamical systems theory also need to be leveraged for improved understanding of brain connectivity.
3. A comprehensive understanding of the structural–functional relationship requires understanding the brain on a macroscale and a deep dive into the microenvironment consisting of genetic, cellular, molecular, microcircuits, and neurotransmitter substrates. Links between micro and macroscale need to be established using computational models and in-vivo animal models. To accomplish this, we need to (1) develop clinically relevant translational animal models of DoC, (2) identify the role of subcortical structures and their interplay with the cortex in heterogeneous models of DoC, and (3) establish the association of cellular (neuronal and nonneuronal) and subcellular (molecular and genetic) mediators with various DoC phenotypes. Effective collaboration between bench and clinical researchers is crucial to allow bidirectional hypothesis testing (i.e., animal to humans; humans to animals)
4. Combining both traditional hypothesis-driven and data-driven approaches is necessary to improve our understanding of DoC. In this regard, there is a need to (1) develop precise theoretical predictions and advance biomarker research to address each dimension of the C-EC-R framework, (2) build a comprehensive set of data and theory-driven biomarkers addressing multilevel analyses, and (3) compare and develop existing theories by promoting adversarial collaboration among thought leaders [41–46] in DoC.
5. There is a need to integrate levels of description, imaging modalities, and theoretical approaches. To develop endotypes, large-scale data sets are necessary to study the heterogeneity of DoC at different levels of analyses (e.g., brain function, structure, phenotype). Such data sets will also facilitate the construction of realistic computational models of DoC and the development of personalized medicine models

### Panel Discussion of the Approach

The panel discussed that the insights into the biology of consciousness are informed by multifaceted research in DoC and agreed that a parallel approach is needed to address these five goals to ensure significant and timely progress. In addition to leveraging existing data sets, there is a need for development of large data sets to help track the current drug therapies used in DoC and to evaluate their efficacy in DoC as means of identifying the underlying mechanisms.

Further, the panel discussed that certain gene activation in cell models may inform the repair, synaptogenesis, and plasticity of genes to help researchers identify therapies for DoC related to those genes. For example, recent studies have revealed that certain genes, such as the human accelerated region genes, are differentially expressed in humans compared with those of chimpanzees and macaques and tend to have a role in expanding cognitive networks (particularly the DMN) [31]. Studies on animal models with the development of validated tools and scales to measure behavior and responsiveness are crucial in understanding the molecular and genetic basis of DoC. The panel suggested that available data on sick behavior (i.e., physical manifestation of illness) and neurophysiology can be leveraged to develop such models while taking confounding factors such as critical illness and systemic injury (leading to physiological alterations such as hypoxia, hypotension, hypothermia) into account.

In terms of the pediatric population, the panel discussed that recovery from brain injury in pediatric patients is unique regarding the fact that pediatric recovery not only means return to premorbid baseline but also involves demonstration of growth along the expected developmental trajectory. Hence, the panel emphasized the need to understand pediatric recovery trajectories as a separate entity from adult cohorts.

Lastly, the panel discussed the need for a central database housing a full list of clinical trials related to DoC and reiterated that database designs that support both hypotheses-driven driven and data-driven approaches in parallel and in a complementary manner is necessary.

### Deliverables

The deliverables for the biology of coma WG include the following:

1. Develop a program project or center of excellence model as the main push to foster open communication with integration of data from animals, humans, and in silico models to effectively move the field forward.
2. Standardize clinical and behavioral assessment in patients with DoC to facilitate effective data collation across centers.
3. Audit prevalence, management, and monitoring strategies and outcomes of patients with DoC in various centers across the world.
4. Undertake health economic analysis for DoC research.
5. Develop standards for clinical and behavioral assessments in DoC.
6. Develop preclinical behavioral, neurophysiological, and neuroimaging models that could subsequently translate into clinical trials.
7. Secure governmental research support for carefully selected individual (RO1 and training grants) as well as research projects (e.g.: PO1, PO3) that integrate across disciplines (preclinical to clinical) and across lifespan (birth to elderly) is essential. Apart from DoC research, funding should also focus on consciousness research as the foundation to understanding DoC.

## Coma Science Database

### Background

A large international data set that captures data from patients with DoC is crucial to gain insights into mechanisms, predictors, and trajectory of recovery and to lay the foundation for future interventional trials. The coma science database WG conducted a needs assessment in 2020 to understand the acute data needs of the coma science community. The majority of survey participants were primarily involved with clinical research affecting patients with DoC. According to the survey, the mission-critical data needed to accomplish goals toward curing coma included patient-specific data, encounter-specific data, and psychometric data. Additionally, survey participants recognized that long-term follow-up data and high-fidelity and time-synchronous data are needed but not currently accessible. The survey results, main research gaps, and challenges have been outlined in the proceedings article from the 1st symposium [21]. A panel discussion at the time concluded that a way forward would include (1) creation of data dictionaries based on common data elements (CDEs), formulation of common language, and terminology as well as data structure framework, and (2) a large simple international database on patients with DoC that is built on CDEs.

CDE development aims to streamline neuroscience clinical research using content standards that enable clinical investigators to systematically collect, analyze, and share data across the research community. The CDE project has been developed in collaboration with the NINDS to create CDEs specific to coma and DoC. A total of ten different WGs have been created to approach a variety of topics, including (1) clinical/behavioral phenotype, (2) hospital course/confounders/medications, (3) imaging (4), electrophysiology, (5) biospecimens, (6) pediatric and other patient groups, (7) therapeutic interventions, (8) physiologic data, (9) outcomes and end points, and (10) goals of care/family data. The WG members were intentionally selected to ensure diversity in scientific expertise, career stage and demographics while ensuring the inclusion of other stakeholders such as patient advocates and industry representatives. Final CDEs are expected to be published in 2022. The intended impact of this foundational work is to (1) reduce time/cost to develop data collection tools, (2) reduce study start-up time, (3) promote data collection in a consistent format, (4) improve data quality, and (5) facilitate collaborations and comparisons between studies and meta-analyses.

Apart from CDE development, there is a need for a central database that would allow us to gain insights into mechanisms, predictors, and trajectory of recovery of coma and to lay the foundation for interventional trials. Currently, there are only a few limited data sets related to DoC with significant gaps, including variability in data collection and fragmentation of data between acute and long-term care. Existing consortia [47–49] offer great experience but also have their own limitations in terms of data diversity and integration. The goal of the CCC coma science database is to be at the intersection of databases focused on (1) scientific discovery, (2) epidemiology, comparative effectiveness, and implementation science, and (3) clinical trials. Based on expert interviews and the needs assessment survey within the scientific community, a set of requirements for the coma science database has been developed. This database is envisioned to broadly capture the needs of all the CCC scientific WGs. A comprehensive coma science database should be able to (1) identify links between structural and functional abnormalities of DoC (2) integrate data across micro and macro scales (3) integrate theory-driven and data-driven approaches (4) facilitate the bridging of bench and clinical research related to DoC (5) facilitate coma research across the temporal spectrum from acute injury to long-term recovery (LTR), and (6) facilitate coma research across the lifespan from pediatric to adult patients. Given the breadth of data required for this project, we need a robust database architecture. Therefore, an ideal database would include the ability to:

1. Accommodate a variety of high-resolution data ensuring data flexibility, data validity and data harmonization.
2. Effectively collect usable data with role-based access into a secure flexible app interface and federated or distributed data structure.
3. Ensure proper data distribution, which includes distribution of primary studies’ data to study teams, exploratory post hoc analyses, performance data for best practices efforts, and patient/caregiver engagement.
4. Ensure data governance and analysis structure to be transparent while maintaining data security, quality, integrity, storage, curation, and portability of a wide variety of data, including biomarker repository.

The coma science database WG has envisioned a complex network of tiered data collection approaches to include small community centers with clinical data to the large academic centers with the ability to obtain advanced imaging and high-resolution physiological data. Additionally, the development of a longitudinal model system to collect data from acute to chronic phases of DoC and beyond would facilitate the conduct of robust outcome studies of DoC. Data from various sources are envisioned to be housed in designated core CCC database centers across the globe, where the data are federated, curated, and distributed to promote DoC research. The WG has identified 50 commercial, academic, and registry-based database enterprises and is actively engaged in conversations with eligible vendors to select the right database for this colossal project.

### Research Priorities for Coma Science Database

To support the development of a coma science database, the WG proposed the following seven priorities:

1. Develop a hybrid database architecture leveraging DoC-specific CDEs focused on linking high-resolution imaging and physiologic data with minimum necessary clinical data, including the long-term outcome data.
2. Design a tier-based network organization to maximize the scalability of high-impact science and clinical trial design.
3. Facilitate implementation of the broader research priorities of the CCC community, spanning scientific discovery and clinical evidence to clinical practice by focusing on concrete, short-term objectives.
4. Facilitate translational scientific discoveries through an infrastructure that enable novel hypothesis discovery and preclinical validation of potential therapies.
5. Create an infrastructure that facilitates longitudinal studies on outcomes assessment and research across the continuum of care.
6. Enable a community of resource for novel statistical, analytical, and methodological approach on big data that emerge from studies on coma science.
7. Design community, patient, and public engagement that provides timely information on best practices, expected outcomes, and available resources.

### Panel Discussion of the Approach

The panel agreed on the need to create a centralized prospective database structure to advance coma science and develop the foundation to support research priorities of other WGs. A comprehensive database accommodating acute through chronic phases of DoC is needed to cater to a variety of researchers across disease trajectory. Big data would be valuable regardless of whether it is collected at centers of excellence in a preplanned standardized format or heterogeneous clinical data acquired in a nonresearch setting, as it allows comparisons across cohorts and can help combine data-driven models of structure and function with theory-driven models. Additionally, big data are needed to test hypotheses, discover therapies and to develop personalized treatment of this complex disorder.

The panel discussed that challenges regarding data collation of existing databases involving DoC include the use of heterogeneous data definitions (e.g., subjective vs. objective data), clinical practice variability, nonstandardized prognostication, and patient/family communication and the variability of management in different facilities across the trajectory of care. In terms of high-fidelity data, specific challenge includes the difference in data sampling frequency across institutions due to the lack of existing standards, thereby limiting data harmonization to the lowest sampling frequency across cohorts. The creation of CDEs and development of standardized protocols are therefore crucial to address these inconsistencies. Patient privacy regulation is another barrier when trying to collate data along disease trajectory from acute, subacute to chronic phases. The panel agreed that innovative ways to address privacy issues are needed to overcome barriers across care settings. Further, apart from serving as the structural unit for centralized data collection, CCC will also serve as the center of excellence for DoC to promote education regarding standardized data definitions, standardized data collection approaches, standardized communication around DoC with patients and families, and the development of guidelines related to DoC.

A comprehensive database approach initiative of the CCC is envisioned to bridge the silos across various stakeholders including preclinical researchers, clinical researchers, nonclinicians, patients, and families and also across the disease trajectory in any individual patient. The panel expressed that a spoke and hub model is likely appropriate for such a massive undertaking with designated cores or centers of excellence for various types of data such as clinical data, biomarkers, physiologic and the imaging data. Finally, in the era of big data, the panel agreed that the integration of artificial intelligence (AI) and machine learning (ML) is important to move the field forward.

### Deliverables

The deliverables for the coma science database WG include the following:

1. Finalization of a vendor to establish comprehensive centralized database and secure adequate funding for initiation and maintenance of such a database.
2. Identification of major hubs or centers of excellence for each data domain (physiologic, imaging, biomarkers, animal data, etc.)
3. Standardization of data collection methodology including standard clinical, diagnostic, and prognostic measures.
4. Development of a process for execution of data use agreements across collaborating centers.
5. Development of education material for dissemination to collaborating centers.
6. Creation of a central database using standardized CDEs that can provide the infrastructure for global collaboration and assist in breaking research bubbles.
7. Seek strategic funding mechanisms with ability to combine databases across modalities (e.g., EEG and imaging) and scientific groups to help foster synergistic collaborations.

## Neuroprognostication of Coma

### Background

History and modern science have taught us that patients who appear unconscious may, in fact, not be unaware. One such example from recent history is that of Saul Bellow, a noble laureate who was assumed to be comatose during hospitalization, yet he was able to provide a stream of conscious narrative from his recollection during the period of presumed comatose state [50, 51]. In a 2006 case report by Owen et al. [7], the investigators used fMRI to demonstrate preserved conscious awareness in a patient who was behaviorally determined to be in a vegetative state. In this study, when the patient was asked to imagine playing tennis or moving around in her home, she was able to activate predicted cortical areas in a manner indistinguishable from that of healthy volunteers [7]. In 2011, Bardin et al. [52] also demonstrated dissociation between observed behavior and cognitive functioning noticed on fMRI. Subsequently, in 2015, Schiff et al. [13] coined the phrase cognitive motor dissociation to describe the general discordance between phenotypic observation at the bedside and the actual state of awareness. In 2017, Edlow et al. [11] reported that covert consciousness is not only present in patients with chronic DoC but also noted in patients with acute brain injury (ABI) in the ICU by using task-based fMRI and EEG techniques. We now know that about 15% of comatose patients in the ICU have covert consciousness and tend to have improved functional outcome at 1 year [16]. Ironically, what science and neuroimaging has come to reveal recently, Saul Bellow had already conveyed to us through his words on a memoir of his illness [53]. Although there is a culture of hegemony of science over humanities, it is important to acknowledge that all the things we face today from climate change and global pandemic to the discovery related to DoC, inevitably involves humanities along with science. Therefore, insights from history, philosophy and literature often provide promissory insights and ignoring such important perspectives could only be considered as wasted opportunities leading to delay in scientific discoveries.

Neuroprognostication refers to an understanding of the most likely prognostic trajectory in patients with neurologic injury by means of biomarkers, neuroimaging, clinical examination and other variables. Accurate neuroprognostication is important for effective communication with the surrogate decision-makers in terms of communicating the potential for recovery, likelihood of disability and the possibility of awakening from coma. During the 1st NIH symposium, five gaps in prognostication were identified: (1) end points for prognostic assessment have not been defined, (2) we do not have standard methods to reliably ascertain and statistically address withdrawal of life supporting therapy (WOLST), (3) we need to standardize and improve diagnostic evaluations and establish the right time window for neuroprognostication, (4) we need prediction tools that use comprehensive clinical information to create statistically appropriate models, and (5) prognostic communication and its impact on families’ decision-making in DOC is understudied.

The existing neuroprognostication research structure of DoC is fragmented, lacks the infrastructure to support collaboration, and lacks prognostic accuracy given categorization of disease based on overt presentation rather than mechanistic understandings. Existing limitations are related to the use of convenience samples with small sample size, lack of blinding and lack of external validation. The biggest limitation, however, is related to early WOLST. There is a need to develop consensus on how to study coma, either by DoC phenotype (coma/stupor, etc.), primary injury type (CA, SAH, ICH, traumatic brain injury [TBI], etc.) or neuroanatomic injury subtypes (brainstem vs. diffuse cortical injury vs. subcortical injury). Neuroprognostication parameters need to be approached as index tests based on relevant neurologic function that are directly related to the functional outcomes and affect the quality of life [54]. There is also a need to understand the role of cortical plasticity, especially in the pediatric population, as return to baseline function may not indicate adequate recovery, especially when developmental milestones are considered over a long recovery period. Given these gaps, the neuroprognostication WG used the American Heart Association (AHA) scientific statement [54] as their template for developing recommendations regarding prognostic indicators. To develop CDEs for high-quality prognostication, the WG proposed that we first develop understanding/consensus on several aspects surrounding prognostication, which include the following:

1. Prognostic markers should not just be available but also applicable and feasible across various clinical settings and disease mechanisms. We need accurate, precise, and clinically applicable tests that can serve most patients after onset of coma.
2. Timing of prognostication is extremely important. Current clinical practice recommendations suggest delaying prognosis for cardiac CA until 72 h and at least 2–3 days for other patients with ABI. However, data suggest that delayed awakening beyond 72 h [55] can occur in up to 32% of patients with CA [54]. There is an urgent need to move away from early prognostic dichotomization to guide interventions. We also need to determine the timing of completion of index test to adequately predict long-term prognosis. In this regard, the WG recommends allowing at least 30 days for neuroprognostication, whenever possible. Use of parcellated approach with stepwise evaluation of milestones throughout the course of recovery is encouraged. Further, the WG proposes delaying cognitive testing for at least 90 days, as acute encephalopathy post hospitalization may significantly alter findings.
3. As coma recovery is a dynamic process, prognostic markers, specific to disease phenotypes should either have the flexibility to be applied across the DoC trajectory or we need to develop multiple tests specific to the stage of coma.
4. Communication strategies congruent with the values and preferences of patients and surrogates should be developed.

The World Health Organization international classification of functioning, disability, and health includes three components: (1) impairments, (2) functional ability, and (3) engagement in activity and participation. A variety of functional and other outcome measures should be included with granularity in prognostic research. Further, to address the numerous confounders of death reporting (e.g., WOLST, extracerebral organ failure), reporting should also include the mode of death, timing from injury, extent of support at the time of death, reason for WOLST and reason for primary injury at the time of presentation (if applicable).

Despite the widespread nihilism, particularly in DoC related to CA, available evidence suggests that patients with coma continue to improve post discharge and outcomes continue to evolve over 1–6 months after injury [56, 57]. Patients ideally need to be out of the hospital to assess outcomes related to complex functional activities. Clinicians need to ensure that the acute symptoms have stabilized and that noncerebral confounders are minimized at the time of prognostication. Efforts should also be made to improve messaging in neuroprognostication guidelines to emphasize prognostic uncertainly given the current lack of definitive evidence. It is very important to avoid all sources of bias and continue full medical support until the right time of prognostic assessment as early DNR orders have been known to result in care limitations [58]. We need a cultural shift to avoid poorly calibrated heuristics or nihilistic opinions by practitioners and work toward developing strict protocols for WOLST with focus on patient and family centric outcome measures. Additionally, as patients may be affected by cognitive, psychological, or physiological impairments, all factors need careful consideration in overall prognostication. The health-related quality of life reporting system should include reported outcomes from both patients and surrogates. Reporting scales should be pragmatic and easy to use and there is a need to work toward validating health-related quality of life for surrogate decision-makers of comatose patients. We should also ensure that all neuroprognostication studies follow the recommended standards of reporting.

In-depth understanding of limitations regarding available evidence for neuroprognostication will guide the design of future studies. Efforts should be focused on developing models with accurate calibration using large amount of clinical data. There are several existing candidate tests and data which need to be evaluated simultaneously with an aim toward development of multivariable models to allow incremental accuracy of neuroprognostication. To develop such models, we need a large multicenter prospective observational coma registry that includes key prognostic data including diverse etiologies of DoC with serial testing over time. Important variables to consider include the following: (1) clinical examination including phenotypes of DoC (2) somatosensory evoked potential (SSEP) to assess thalamocortical connectivity, (3) serological/cerebrospinal fluid/chemical/genetic biomarkers, (4) Functional neuroimaging pertaining to stages of coma, (5) EEG with quantified parameters, and (6) brain-computer interface and other novel technologies. Additionally, inclusion of baseline variables (age, race, ethnicity, zip code, comorbidities, lifestyle, frailty, social support network, socioeconomic status, etc.), intensity of management (acute and rehab phase) and inclusion of pediatric population is important. Also, there is a need for innovative methods of analysis to assess predictors from early to late phases of DoC. Accurate modeling of prognosis and disease trajectory for personalized prognostication could benefit from advanced statistical methods and novel tools such as ML technology for data assimilation. In such models, external validation of the outcome metrics in a large independent cohort is crucial.

### Research Priorities for Neuroprognostication

Based on the identified gaps, the WG formulated four main research priorities for neuroprognostication:

1. Develop prognostic indicators for patients with various mechanisms of DoC meeting consensus criteria that is accurate, reliable, conducted at specific time points, flexible across the disease trajectory, and congruent with patient and surrogate’s preexisting values and preferences.
2. Develop consensus on how to study neuroanatomic injury subtypes and assessment with targeted modalities linked to meaningful outcomes (including impairments, functional ability, engagement, and participation).
3. Design a multicenter prospective observational coma registry including pediatric population with data captured across the outcome trajectory (i.e., age-based measures of recovery).
4. Identify ways to facilitate communication of prognosis and prognostic uncertainty between clinicians and patients/families.

### Panel Discussion of the Approach

The panel discussed that there is a general sense of nihilism among clinical practitioners and one of the goals of CCC should be to help communicate the complexity of DoC prognosis and help develop an understanding of the confidence interval for neuroprognostication at various stages of DoC. A parcellated prognostication approach with stepwise milestones throughout the course of recovery would be more appropriate than a dichotomized prognostication of favorable versus unfavorable outcome. Panel reiterated the complexity of neuroprognostication in the pediatric population due to the continued neurodevelopment through the course of recovery and highlighted the need for robust longitudinal studies on recovery trajectory and prognostic approaches on all age groups.

The panel also discussed that one of the issues with clinical trials on neuroprognostication is the ethical aspect of continuation of life supporting therapy especially if it does not concur with the values of the patient and surrogates. Such patients and families should be excluded from clinical trials on prognostication. Widespread education endeavors are needed to change the culture of nihilism among providers and families. However, a trial that requires prolongation of DNR or WOLST wait times would require an in-depth discussion with the families in addition to detailed informed consent. Data from countries with cultures that support prolongation of life supporting measures would be helpful in determining the natural history of DoC. Within the United States, one of the limiting factors might also be related to the DNR orders in which some clinicians and families may perceive this to mean “do not provide aggressive medical therapy,” even though the intended use of this order is only for the clinical management of one specific clinical scenario (i.e., CA). The panel highlighted the need to develop protocols surrounding WOLST decisions and address confounders (e.g., sedative medications, systemic injury, etc.) that impact outcomes. Further, the panel discussed the need to identify key elements and strategies for communicating uncertainty in a tactful, emotionally supportive and culturally competent manner followed by verification of surrogates’ understanding of outcome communication.

### Deliverables

The deliverables for the neuroprognostication WG include the following:

1. To define consensus standards for prognostication research: The goal is to define the highest value prognostic tests with an appropriate timeline for testing that is needed to determine patient/family centric high value outcomes.
2. To develop mechanisms for natural history and comparative effectiveness studies for neuroprognostication.
3. Develop mechanisms to study modifiable barriers to family/team communication that impedes practice of appropriately delayed prognostication and decision-making.
4. Acquire funding for both network infrastructure and individual investigator-initiated studies to advance neuroprognostication in patients with DoC.

## Care of the Coma Patients

### Background

To cure coma, we need to provide effective care for our patients with DoC. Care involves two aspects: (1) caring about a patient, which is the emotional aspect, and (2) caring for a patient, which involves a set of actions or interventions performed to meet the patient’s needs. Caring for the patient is integral to CCC for three main reasons (1) if the comatose patient dies before curative intervention (i.e., if the patient does not receive the care), the possibility of recovery is irrevocably eliminated; (2) identification of variables of care that confound the benefit of clinical intervention (e.g. WOLST within the first few days) can help reduce sample size needed for future studies measuring the effectiveness of the curative interventions; and (3) it is essential to identify interventions that will move the patient toward (benefit) or away (harm) from recovery.

The care of coma patients is highly variable. Routine clinical assessment and monitoring are tailored according to the availability of the tools and technology, the culture of the health care setting, clinical expertise, and experience of the health care providers. To date, there are no global standards, even for basic tasks such as examining patients with DoC. Although there are new DoC care guidelines promoting use of advanced DoC biomarkers, these guidelines point more toward timing in the subacute to chronic phase, and have no clear recommendations for the acute phase. In terms of therapeutic intervention, the use of neurostimulants in patients with DoC is also highly inconsistent. We have not yet established the optimum timing for clinical interventions such as tracheostomy, nor have we gained an adequate understanding of optimum frequency and duration of physical, occupational, and cognitive therapy to achieve the best possible recovery. This variability in care leads to heterogeneity in patient outcomes and introduces bias in DoC research.

Apart from inconsistency in care, variability is also present in the type of care providers. In day-to-day clinical practice, care of the patients with DoC is provided by multiple caretakers, including physicians, nurses, therapists, pharmacists, social and spiritual services, and family members. For every intervention, it is important to assess behavioral or physiologic cues from the patient to ensure the appropriateness of any given intervention. As an example, although bathing and cleaning is a routine part of clinical care, if the intracranial pressure appears to be on an uptrend in a patient with ABI, it would be reasonable to treat the elevated intracranial pressure before lowering the head of the bed for cleaning. Such judgments regarding optimum timing of intervention based on patient cues have been described as the cue-response theory [59] and should be implemented in routine clinical practice.

The WG has identified six main domains impacting care based on the observed gaps. Broadly, these domains include the patient level, provider level, and system-level factors that interface with each other and influence the patient’s care. Existing gaps along these six domains include the following:

1. Assessment and monitoring: (1) Available assessment tools have substantial variation and often have poor psychometric properties; (2) feasibility, necessity, and ethical considerations of advanced monitoring (e.g., invasive intraparenchymal monitoring tools) have not been well studied; (3) there is a need to differentiate and delineate routine vs. advanced monitoring and understand the impact of these tools on patients outcomes; (4) objective assessments should replace subjective assessments to decrease variations; and (5) Patients and family experience should be integrated into assessment tools across care settings and through transitions of care.
2. Timing of intervention: (1) We need to identify optimal timepoints for routine care interventions (e.g., should the intervention occur at a singular or serial time points?); (2) we need to assess the effectiveness of time points for interventions across various DoC etiologies (e.g., if a therapy at a certain time point is effective in one etiology, would it directly translate to other etiologies across the spectrum of DoC?); and (3) we need to establish the optimum duration of routine care interventions.
3. Expertise and experience: We need to identify expertise and interdisciplinary team needed across care settings for specific bedside assessments and interventions to avoid variability in care. This may be achieved by determining desired training/certification of designated experts for specific assessments and interventions throughout the care continuum.
4. Technology: (1) we need to determine the clinical utility of modern technology and their impact on patient outcomes; (2) we need to identify optimal mechanisms to implement useful and helpful innovations across care settings feasibly and effectively; (3) we need to determine the cost–benefit ratio of care technologies and determine the impact on patient and health system outcomes; and (4) we need to determine the limitations and benefits of technological advances including AI-based tools, data visualization tools, automated emergency alerts, adapted gaming for therapeutic and educational purposes and the feasibility of nontraditional data types.
5. Religion and culture: (1) We need to understand and incorporate family views regarding faith and spirituality in the healing process; (2) we need to be cognizant of religious and cultural norms surrounding WOLST; (3) we need to establish and evaluate models of care delivery that integrate family into all decisions throughout the continuum of care; (4) we need to consider legal aspects of WOLST including cultural/ethical/religious preferences, family and patient values and beliefs, use of surrogate decision-makers; and (5) we need to consider treatment bias related to spirituality or religion of clinicians as well as access to religious advisors in the care setting (impact on communication, prognostication, and situational debriefing).
6. External and regulatory factors: (1) We need to consider the impact of regulatory requirements and guidelines on local resource allocation and outcomes throughout the continuum of care; (2) we need to minimize variability in care, (3) we need to move toward a centers of excellence that provides expertise and recommendations throughout the continuum of care; (4) we need to determine the positive vs. negative impact of program certification on outcomes as well as the resource strains and burden on participating organizations; and (5) we need to determine potential benchmarks and quality indicators specific to the care of patients with DoC across treatment settings.

### Research Priorities for Care of the Coma Patients

These six domains are interconnected within the clinical implementation, public health, and clinical research aspects. Addressing the domains through scientific innovation and discovery will inform the appropriate clinical intervention to improve the care of patients with DoC. The WG proposed nine overarching research priorities based on the identified gaps:

1. To establish validated and objective measures for the diagnosis of DoC that is consistent across patient populations, disciplines, treatment, and resource settings and measured at a predefined interval from index event.
2. To establish global incidence, prevalence, and etiology of coma, the natural history of disease progression, and impact of current practices on acute post/acute outcomes and resource allocation
3. To standardize coma assessment battery throughout the continuum of care to provide consistent approach to clinical examination, indicators for advanced imaging, and better accuracy and consistency of prognostic models across settings and populations.
4. Identify the current degree of variations in practice and impact on patient acute and postacute outcomes.
5. Establish new best practices, reinforce existing practices, and eliminate ineffective practices and timing for assessment, monitoring, treatment, and care transitions.
6. Minimize practice variations to ensure consistent and effective evidence-based coma care across resource settings.
7. Routine integration and examination of consciousness-promoting therapies in acute care using pragmatic trial designs.
8. Implementation science to identify optimal strategies for integrating identified best practices into routine clinical care globally.
9. Availability of resources and costs associated with long-term coma and DoC management for patients and families.

The WG emphasized the need for a pragmatic approach to advance care of the patients with DoC with the appropriate understanding of global disparities in care and regional cultures and practice preferences. The group highlighted the need for close communication and collaboration between preclinical and clinical research teams to create a highly functional and cohesive research group. Data repositories should include current clinical practices across DoC etiology and across the spectrum of care so future research can appropriately account for these variations while actively standardizing practice where possible.

### Panel Discussion of the Approach

The care of the coma patients is the center of the CCC mission as, without proper care, there is no cure. The panel members discussed that many existing interventions and treatments for DoC are currently based on common or best practice standards that lack strong evidence for generalized application, especially in the pediatric population. This has led to heterogeneity in care, which needs to be recognized and promptly addressed using education, research, and implementation of evidence-based practices. Variability in practice makes it difficult to test the efficacy of any intervention that might be helpful in curing coma, therefore, standardized approaches increase our chances of finding a cure. The panel also highlighted the need for widening the search for funding sources to support the interdisciplinary research teams that focus on the various needs of the multidisciplinary care teams working with patients with DoC.

The panel expressed that while working toward reducing variability and optimizing care of the patients with DoC, several factors at the patient, clinician, and system level should be considered. For example, while advocating for standardized practices regarding WOLST decisions, we need to consider patient and families’ culture and values, prognostic humility, ethical considerations for clinicians, and resource needs at the system level, including facility cost of long-term care for patients with DoC. Extracting natural history data from countries and regions that are culturally supportive of prolongation of life-sustaining therapies may be one approach for obtaining such data. In terms of standardization and protocolization, the cost/benefit ratio of new and additional regulatory oversight implementation should be considered. Additionally, the CCC should seek ways to work within cultures that support the individual clinician’s ability to critically evaluate decisions of care, as strict regulations and protocols that demand compliance over clinical judgment can often be obstructive. It is also important to heed the needs of the clinical team that cares for patients with DoC. One such need is to develop ways for streamlined communication between multidisciplinary care teams with the ability to freely discuss and integrate care decisions.

In terms of integration of novel tools and innovation, the panel expressed the need to understand the limitations of such tools. For AI-based tools, it is very important to understand the models on which the tool was tested and validated as the dictum “garbage-in-garbage-out” holds true, especially in in silico models that cannot capture the variables and confounders in a real clinical setting. A model complex enough to identify which changes are significant and how they affect the patients does not yet exist. Large volume of prospective data with models including overt and covert clinical variables pertaining to routine clinical care are needed to improve the reliability of AI in critical care settings. Although care of the coma patients includes optimization and standardization of sophisticated measures such as electrophysiological, biophysical, and other biomarkers as well as innovation such as AI, approaching the low hanging fruits such as standardization of basic approach for evaluation of comatose patients can be impactful. In the current clinical setting, simple care aspects such as the variability in the basic assessment of the comatose patient (e.g., pupillary assessment using a penlight, vs. divergent phone light vs. pupillometer or motor examination using sternal rub or trapezius pinch vs. extremity pinch) can be significantly limiting in measuring impact of our clinical interventions. Furthermore, the panel highlighted that care of the coma patients is complex, and one-size-fits-all model is inadequate given the multiple factors specific to each clinical scenario, including variation in care teams based on pathology. However, despite this heterogeneity, identifying common grounds is possible on some of the key factors, and efforts should be focused on improving and standardizing common aspects of assessment, monitoring, and therapy.

### Deliverables

The deliverables for the care of the coma patients WG include the following:

1. Identify variations in care and related impact on outcomes across resource settings and populations.
2. Identify existing and establish new evidence-based best practices to minimize variations in assessment, timing, monitoring, technologies, and treatments for coma care.
3. Identify optimal strategies using an implementations science approach to integrate established best practices in clinical settings.
4. Create models of care that integrate family engagement and decisions making throughout the lifespan.

## DoC and General Anesthesia

Preceding the discussion related to early clinical trials, cortical effects of anesthetics were reviewed. General anesthesia (GA) leads to a drug-induced, reversible state that consists of antinociception (i.e., loss of nociceptive processing), unconsciousness, amnesia, akinesia (i.e., loss of movement), and maintenance of physiological stability [60]. Numerous studies in humans and animals under GA have informed the study of consciousness. In 1935, Frederic Bremer performed the well-known brain stem intercollicular transection which he called the cerveau isolé preparation [61]. When he transected and separated the brainstem from the spinal cord, he noted an “awake appearing” EEG pattern, whereas when he transected the brainstem from the cortex, he noted “slow delta oscillations” mimicking slow-wave sleep. This and subsequent studies demonstrated that intact brainstem is necessary to maintain the state of arousal. Studies using GA also demonstrate the slow delta oscillation pattern [62]. Slow oscillations are a neural correlate of propofol-induced anesthesia, indicating a shift to cortical dynamics in which local neuronal networks remain intact but become functionally isolated in time and space [63]. Hence, models of propofol anesthesia have the potential to provide tremendous insights into the biology of coma. Unlike propofol, recordings during ketamine anesthesia show alternating high frequency (Gamma) oscillations and oscillations that are coordinated across the cortex from frontal to occipital regions [64]. At low doses, however, ketamine primarily only acts in the inhibitory interneurons leading to fast oscillations. On the other hand, nitrous oxide produces profound slow oscillations that are transient and is likely related to inhibition of glutaminergic pathways arising from the brainstem [62]. Dexmedetomidine’s action is closest to sleep as it blocks the release of norepinephrine coming from locus coeruleus thereby inhibiting input from brainstem to thalamus leading to slow oscillations. In low doses, it produces sleep spindles (mimicking stage II non-REM sleep), whereas high doses with deep sedation generate slow oscillations (mimicking stage III non-REM sleep) [62, 65].

The mechanisms through which anesthetics produce their neurophysiological responses in cortical, thalamic, and brainstem circuits differ by drug class, patient’s age, and physiological state. Slow delta oscillations are also noted to be predominant in very young (0–3 months old) [66] and very old patients anesthetized with GABAergic drugs such as sevoflurane, likely due to poor development (in 0–3 months) of thalamocortical connections or the disintegration (old population) of thalamocortical connections. Similarly, patients with systemic sepsis and those on chemotherapy also demonstrate predominant slow oscillations. In summary, all anesthetics produce slow delta oscillations, most likely through inactivation of brainstem projections to thalamus and cortex and through thalamic and cortical hyperpolarization.

## Early Clinical Trials

### Background

To cure coma, we need a multifaceted approach. Ideally, we need (1) the ability to map human brain networks that are essential for consciousness and allow for repeated bedside assessments in the acute phase; (2) new tools to identify preserved brain network connections that allow personalized connectome mapping; (3) to develop point-of-care fluid biomarkers to identify patients who may benefit from therapy and have valid metrics for assessment of treatment response; (4) to develop targeted treatments personalized to patients; and (5) to ensure long-term follow-up.

One of the main goals of the CCC is to determine how early interventions may offer novel opportunities for consciousness recovery through clinical trials of consciousness-promoting therapies. Patients with DoC are often overlooked in clinical trials, especially in the acute phase of the disease. Early interventions could offer immense opportunities for consciousness recovery. Development of such early consciousness-promoting therapies in the ICU could (1) prevent premature WOLST; (2) facilitate recovery of consciousness; (3) decrease ICU-related complications; (4) improve opportunities for rehabilitation; and (5) minimize long-term disability.

As part of the CCC mission, the early clinical trials WG is charged with developing proof-of-concept clinical trials that improve the accuracy of prognostication as well as treatment strategies that promote recovery of consciousness throughout the continuum of DoC care. The overarching goal of the WG is to:

1. Develop trials based on defined patient phenotypes and endotypes.
2. Develop adaptive, exploratory interventional trials to identify interventions that support the recovery of consciousness.
3. Develop simple master protocols to achieve these deliverables and
4. Implement findings into routine clinical care.

To navigate these aspects, there is a need to develop innovative ways to categorize various types of DoC to accurately monitor treatment response [23]. Adoption of successful approaches related to other diseases could be helpful. For example, acute respiratory distress syndrome is similar to DoC as a condition that broadly captures various mechanisms of lung injury culminating into one type of overt clinical presentation. Investigators in this field have noted that treatment response for acute respiratory distress syndrome is dependent on hypo or hyperinflammatory disease subphenotypes [67]. Categorization of DoC in a similar fashion might be useful to study treatment response. We also need to consider the appropriate timing of outcome prediction and integrate key outcome measures proposed by the CDE WG. Development of surrogate outcomes measures to determine meaningful clinical outcomes is useful. Moreover, the infrastructure and design for the early clinical trials need to be defined. Integrating successful aspects of existing networks such as the Strategies to Innovate Emergency Care Clinical Trials Network (SIREN) [68], Transforming Research And Clinical Knowledge in Traumatic Brain Injury (TRACK-TBI) [69], and the NIH StrokeNet [70] with “model systems” approach might be a reasonable consideration for DoC infrastructure. In terms of research design, there is a need for observational/natural history studies as the foundational step. Adaptive trials where intervention can be modified based on response should be considered whenever possible. Traditional randomized control trials with development of biomarkers [71] should follow these initial efforts.

The CCC Coma Epidemiology, Evaluation, and Therapy [72] survey recently conducted an international cross-sectional survey to assess (1) variability in coma definitions; (2) common etiologies, assessment tools, and treatment strategies; and (3) attitudes toward prognosis. In this survey, a total of 258 health care professionals working with coma and DoC completed the survey, out of which 83% were physicians with majority from academic or teaching hospitals in urban areas. The survey results revealed that the most common diagnostic tools used to assess coma patients included neurologic examination (98%), basic neuroimaging (89%), and noninvasive EEG monitoring (94%). Variability in clinical assessment was also noted with the use of various tools such as GCS, neurological examination, National Institute of Health Stroke Scale (NIHSS), Confusion Assessment Method for the Intensive Care Unit (CAM-ICU), Full Outline of UnResponsiveness (FOUR) score, CRS-R, and others across institutions. Respondents identified their top three future areas of coma research, with high interest expressed for treatment, prognostication, pathophysiology/mechanisms of coma, and diagnostics. In a global platform such as the CCC, heterogeneity in patient population and clinical practice is inevitable. However, this variability could be utilized for comparative effectiveness research. For example, heterogeneity in patient population can be utilized to explore various phenotypes and endotypes and to identify minimum common denominator of anatomy and physiology across diseases that lead to DoC [23]. Similarly, DoC cases with spontaneous recovery can be used to understand natural history of coma.

Efforts across the CCC have created opportunities to bridge siloed efforts and leverage the expertise of diverse groups spanning the continuum of care to provide a big picture of consciousness research. Regardless, the charge is complex with no precedent to follow and has numerous roadblocks to overcome. To start, we need to address some basic questions related to early clinical trial in DoC such as:

1. Which patients are likely to respond to treatment?
2. Which trial designs are most efficient?
3. Which outcome measures have the best sensitivity and specificity?
4. Which is the best approach to control for variability of care?

### Research Priorities for Early Clinical Trials

Based on the gaps, the WG proposed six research priorities:

1. Set expectations for future trials (define what good research looks like).
2. Assess current variabilities in care and trial resources across collaborating sites.
3. Develop proxy biomarkers that allow for trials with small patient numbers to inform larger trials on timing of treatment and promising strategies. Additionally, develop a consensus on the specific clinical measures (GCS vs. CRS-R), imaging tools, blood/CSF biomarkers, electrophysiology and the pharmacodynamics.
4. Define end points, ways to assess them, and identify the best performing measures. For example, should the trials use traditional Glasgow Outcome Scale-Extended (GOSE) versus functional measures? What is the appropriate timing of measurement? Should the outcomes be measured short term or long term? Regardless of the outcome measure selected, it should be feasible and patient/family-centric.
5. Determining best infrastructure and designs for trials.
6. Enrich samples based on similarities among patients.

### Panel Discussion of the Approach

The panel discussed that sample enrichment with proper sample selection is important to ensure success of the clinical trials [73]. Enriched samples could improve efficiency, reduce cost, minimize side effects, and shift the risk/benefit ratio of any given intervention. Patient selection based on phenotyping and endotyping with the help of imaging, electrophysiology and behavioral measurements is important to yield efficacious results [74–78]. With the advent of new tools in the field, we have the ability for nuanced, rigorous and detailed neurologic assessments that allow us to enrich patient samples. Development of standardized assessment tools in the ICU would be a good starting point to improve sample enrichment. However, we also need to be cognizant of selection bias. In the acute period, given the multimodal monitoring environment of the ICU, use of automated data collection and assimilation would be highly useful in handling big data in patients with DoC. For the outcome studies, granular outcomes that are meaningful to patients and families should be included [79]. Additionally, as patients continue to recover over months to years, longitudinal studies measuring long-term outcomes beyond 3–6 months should be included [80, 81].

To begin, we first need to identify key site investigators across the CCC research network, and work on the logistics of interventional fidelity as we move forward with adaptive trials. However, the panel reemphasized that given the need for long-term follow-up to assess DoC outcomes, identification and validation of early biomarkers to evaluate treatment response and early prognostic markers would help determine go/no go decisions, especially for high-fidelity adaptive trial designs. Rather than a sequential approach, parallel but noncompeting studies with slightly different inclusion criteria for DoC should also be considered to lessen the screening burden on the local sites. One of the biggest gaps impeding design of supervised ML models or adaptive trial designs is our limited understanding [82, 83] of the natural history of DoC. Therefore, understanding natural history is an important foundational step along with understanding of the major confounders affecting outcome.

### Deliverables

The deliverables for the early clinical trial WG include the following:

1. Understanding the breadth of heterogeneity in care.
2. Developing intermediate endpoints using surrogate measures such as biomarkers.
3. Identifying optimal patient and family centered outcomes.
4. Tiered approach or program of research using various study designs.

To accomplish this, the WG proposed a 4-tiered approach:

- Level 1: Global point-prevalence/prospective cohort study (limited data [Electronic Medical Record]+ and hospital discharge outcome that will help assess variability of practice.
- Level 2: Prospective observational study. Term-limited recruitment with specified follow-up time points to help us understand biology and recovery.
- Level 3: Comprehensive model system with continuous enrollment of patients, lifetime follow-up.
- Level 4: Prospective trials. To address specifics aims related to diagnostics and therapeutics.

The WG also outlined a 5-year roadmap for early clinical trials:

- Year 1–2: Definition phase: focused on establishing definitions, aims, standard expectations and assessments. We are currently in this phase.
- Year 2: Design/Development Phase: Design right trial for specific questions. Estimate sample size and cost. Focus on subject selection and outcome measures. This phase will focus on prospective cohort studies (basic and advanced) and identify biomarkers and surrogate endpoints.
- Years 3–5: Implementation Phase: Initiation of clinical trials.

## Long Term Recovery

### Background

According to Confucius, “study the past if you would divine the future.” In order to understand the LTR potential, we must study those who have demonstrated what is possible. Taking this long-view provides a perspective from which to study LTR and (1) define prognostic endpoints; (2) understand personal or environmental factors that influence good long-term recovery; (3) understand and implement interventions that support and optimize long-term recovery; and (4) understand health care disparities.

The current state of LTR research can be summarized in the following points:

1. Substantial recovery over long periods of time is possible for a sizable group of individuals with prolonged DoC.
2. Individuals with TBI tend to fare better than non-TBI (nTBI) with respect to DoC. Improvements over a 10-year period have been reported in TBI, with > 25% living without in-home support and are able to work in a competitive work environment. In addition, there are known improvements documented over 2-year period in patients with nontraumatic causes of DoC [84].
3. Comorbidities and secondary conditions make recovery course nonlinear and heterogeneous from a statistical perspective.
4. There are very limited studies in children, but studies have documented recovery up to 2 years post-injury.
5. Most DoC studies are derived from inpatient rehabilitation cohorts, that represent minority of DoC population who could benefit from this type of rehabilitation care, yet they do not receive this specialized care [85].

Some recent work within the TBI Model Systems network can serve as an exemplar for LTR studies. The TBI Model System has the infrastructure to follow thousands of survivors over the lifetime to observe the natural history of the disease. In one such study, the investigators looked at 2058 patients with TBI and DoC at the time of rehabilitation admission and found that 82% recovered consciousness during acute inpatient rehabilitation and 40% became partially or fully independent [85]. Absence of intraventricular hemorrhage and intracranial mass effect has been associated with recovery of consciousness during rehabilitation along with young age and male sex as markers of improved functional outcome. The TBI Model Systems has also been beneficial in informing long-term outcomes. Previous work has shown that 3.3% of patients with TBI die by 1 year and by 5 years, 74% of those with DoC at discharge become conscious, 19.6% are able to live without in-home supervision and notably 18.7% are competitively employed 5 years post-injury [86]. By 10 years, there are additional measurable gains in independence particularly for those who began to improve later in their course of recovery [80]. While such findings are very important to shed light on the potential for recovery after coma, it is essential to realize that the TBI Model Systems only focuses on a small subset of patients with DoC (i.e. TBI) and that, many patients with DoC who may benefit from such long-term rehabilitation may either undergo WOLST or never get the opportunity to rehabilitate to achieve optimal outcome. Government mandates to provide specialized care to all patients with DoC who need such care, could improve accessibility of care.

The WG identified several key steps to address the many existing gaps on DoC LTR research [87]:

1. An increase in knowledge base about recovery trajectories in the absence of limitations to care (i.e., WOLST). Such knowledge is important to:

- Generate scientific evidence that supports guidelines for WOLST.
- Identify and communicate key elements and processes around uncertainty with DoC recovery.
- Development of more accurate and flexible modeling of recovery trajectory using advanced statistical methods, widely adopted and implemented CDEs, and patient-centered outcomes.
- Utilization of data sets that track patient data across the continuum of care using patient-centered outcomes that can be integrated into clinical practice and workflow.
- Enhanced understanding of biomarkers and mechanisms affecting LTR course.
- Integrated research/health care delivery systems that translate novel research treatments and technologies into practice and facilitate access to DoC care programs across the recovery continuum.

### Research Priorities of LTR WG

Rehabilitation is a foundational component of studying and optimizing LTR trajectory. The NIH 5-year research plan on rehabilitation developed in 2016 outlines priorities in multiple areas of rehabilitation medicine and research that can benefit individuals with temporary or chronic limitations in function [88]. This proposal highlights six research priority areas and emphasizes the need to coordinate efforts across other federal agencies. The NIH priority includes (1) promotion of rehabilitation across the lifespan to track factors that cause disability and common secondary conditions that occur over time as well as to identify health disparity that occur across the life span; (2) prioritization of community and family given the impact of caregivers and community on patient outcome; (3) technology development by promoting interdisciplinary collaboration within the health disciplines and with colleagues in computer science, math, statistics, engineering, and the end-users of the technology; (4) development of new methods and designs for research with emphasis on data sharing and knowledge translation; (5) promotion of translational science in rehabilitation research to advance understanding of biological, physiological and behavioral mechanisms that underlie DoC recovery and adaptation through precision medicine, animal models and genomics; and (6) building research capacity and infrastructure through training, consultation, and collaboration to develop basic scientists, physician scientists, allied health professionals, and engineers who focus on biomedical devices or rehabilitation.

These rehabilitation research priorities of NIH [88] align with many priorities of the LTR WG and fit within the context of a DoC multisite network. Based on the areas of intersection, the LTR WG proposed the following priorities:

1. Rehabilitation across the lifespan: Strategies to promote rehabilitation across the lifespan include: (1) identification of preexisting LTR cohorts and using them to understand the impact of early withdrawal of life supporting therapy (WOLST) as well as to develop communication strategies for conveying prognostic uncertainly regarding natural course of DoC; (2) utilization of CCC LTR experts and existing CCC sites for biomarkers studies using CDEs specifically designed to capture the LTR perspective; (3) application of DoC-specific CDEs across the disease continuum at all consortium sites and promoting pragmatic follow-up batteries utilizing novel technology such as telemedicine and wearable devices; (4) identification of appropriate LTR assessment tools with consideration for metrics sensitive to technologies and treatments; (5) identification of appropriate timing across the course of recovery for operationalizing and implementing specific rehabilitation strategies. For example, integrating physiatry in post-acute phase to manage specific issues such as delirium, spasticity, dysautonomia, etc. and early neurostimulation as standard of care in the acute post-injury period; and (6) integration of variables related to social determinants of health (e.g., race, ethnicity, economic disparities) to evaluate their impact on LTR.
2. Longitudinal studies to assess recovery trajectories: We need to evaluate prognostic accuracy for LTR as premature WOLST is quite prevalent. Development of prediction models would be helpful to guide clinical decisions. Inclusion of physiatry colleagues in LTR research is critical. Several existing databases and networks for adult recovery-based research on various DoC etiologies such as TBI [89–92], CA [93–95], and others [68] must be leveraged to expand our understanding of LTR. Such existing database and registries could help construct robust generalizable models to predict long-term outcomes, facilitate studies with propensity score matching of DoC cohort with or without maximal medical therapy and plan future multisite prospective observational studies.
3. Utilization of existing databases and networks for pediatric/Neonatal recovery-based research: Similar to the adult registries, leveraging existing pediatric and neonatal databases [96, 97] and establishment of cross-collaboration across research groups is essential to advance pediatric research. Pediatric research in DoC is limited by the overall small volume of patients compared with adults. Given the variability in DoC etiology, range of development across the pediatric age group and pathobiological variations, the overall low number of pediatric patients with DoC pose unique challenges in identifying pediatric DoC phenotypes and endotypes. A phased approach starting with increased awareness of pediatric DoC concepts, establishment of CDEs and consensus in outcome measures followed by harnessing parallel efforts through centralized initiatives of CCC modeled after successful precedents such as the NIH brain initiative [98]; can drive the development and execution of proof-of-concept clinical trials with plan for subsequent prospective observational functional outcome and biomarker studies.
4. Technology development and use: Important considerations for technology development include age and stage of development; brain health, secondary conditions, and comorbidities, environmental milieu including barriers to use, health disparities, infrastructure and access, utility, cultural acceptability, average caregiver’s ability to utilize the product as well as family/caregiver education and the availability of economic resources for repair and maintenance. As for prioritization of technology, since portability is extremely important to patients and caregivers, innovation related to motor assistance with focus on ergonomics is crucial. Similarly, regaining fine motor movements that facilitate integration into the community and work environment equates to economic independence for adults and meeting educational expectations for pediatric patients. Hence technology focused on improving mechanical precisions using technology such as the virtual reality should be considered. Cognitive training is equally important with the development of age and stage-appropriate content for adequate cognitive rehab. Additionally, augmented communication tools using brain-computer interface can significantly enhance quality of life of patients and caregivers and should also be prioritized. As new innovative tools continue to develop, equality in access to care should be a key consideration to ensure that these initiatives do not expand the existing gap in health care disparity [99]. The WG suggested a tiered approach to technological development and innovation. Such approach could include assessing existing technological capacity and capability in phase 1, building collaborations across centers with similar technology and rehabilitation protocols in phase 2 and ultimately designing new technology-based rehabilitation programs in phase 3.
5. Translational Science: The panel noted that the next area of research prioritization is biomarkers of LTR for determination of prognostic trajectories. Investigators should strive to characterize longitudinal profiles as the initial step toward population stratification in assessing treatment response and prognosis across age groups and recovery continuum, with the understanding that recovery is not a linear process. Biomarkers that capture systemic response to neurological insult (e.g., factors contributing to prolonged hospital course or systemic complications) should be considered. Markers that capture both adaptive and maladaptive responses associated with repair and recovery are important, such as the structural plasticity (neurogenesis, cortical reorganization, synaptogenesis, axonal sprouting), functional plasticity (cognitive/experiential supported processes of learning and memory) and maladaptive plasticity (epilepsy, neuropathic pain, incomplete motor recovery, spasticity, etc.). Further, it is important to account for personal biology, etiology of DoC and complications as that can have major impact on LTR. Biomarkers focusing on secondary conditions after brain injury (e.g., depression, cognitive impairment) should also be considered. Finally, there should be appropriate stratification and power calculation to capture racial differences in biomarker functionality, profile, and utility.

The three main components for LTR biomarker research include: (1) neuroimaging for detection of covert cognition (event related-fMRI), functional integrity of consciousness (resting state-fMRI), metabolic evaluation (Positron emission tomography/PET) and monitoring of secondary brain damage (Diffusion Tensor-MRI); (2) neurophysiology for monitoring of paroxysmal activity (EEG), restructuring of sleep stages (polysomnography), integrity of the sensory paths and late potentials (Evoked response potentials); and (3) wet biomarkers for chronic inflammation, cell migration, hormones and neurotrophin markers, CNS exosomal markers as well as genetics and epigenetics. Biomarker discovery requires a phased approach with (1) designing data-driven models to learn from the existing databases of patients with DoC using big data and ML technology; (2) identification of potential new biomarkers and strategies to harness data-driven evidence to guide rehabilitation; and (3) developing a consensus for the routine use of the most informative long-term biomarkers.

6. Models of DoC care. Models of DoC should follow a continuous care pathway supported by best evidence with embedded data collection that informs current care while also contributing to research and future care. As a guidance for creation of such a model, the CCC can study the chronic care model that leverages the interplay of health systems (delivery system, clinical information systems and decision support systems) and the community in which one lives (public policy and resources, supportive environment, self-management support) to achieve the best outcome possible by incorporating various components of these two integral systems [100] to provide people with adequate resources and public support for optimum health and recovery. The model of DoC should comprise six major elements that include (1) respect for patients’ right for autonomy and self-determination; (2) incorporation of clinical trials into health care delivery; (3) facilitation of high-quality data collection; (4) platform for progressive prognosis conversation; (5) maximization of contribution from clinical teams; and (6) accommodation of patients with diverse needs. An example of a successful model includes the NIH model of designated cancer centers.

In summary, we need to develop valid and effective value-based care paradigms to, improve quality of care, increase access to care, prevent costly complications, detect changes in trajectories that require modifications of care, develop mechanisms to collect data, share data and have communication mechanisms in place to deliver collaborative care. Such a model would then allow us to provide the care that could help improve outcomes in patients with DoC.

### Panel Discussion of the Approach

The panel discussed that we currently lack meaningful data on patients with DoC from vulnerable populations with regards to age, gender, and ethnicity. There is a need to focus on a large-scale population study that includes vulnerable populations to adequately represent these groups into the data sets with derivation of relevant outcome measures.

The panel agreed that various LTR priorities align with NIH priorities along the domains of longitudinal studies, translational biomarkers, integration of technology and models of multidisciplinary integrative care. Although DoC affects millions of patients worldwide and is a major source of morbidity and mortality with high economic impact, adequate funding is lacking in this field of research. Panel agreed that with appropriate funding, CCC is well-suited to spearhead this mission to not only leverage existing infrastructure such as the NCRC, global collaborative networks and Neurocritical Care Foundation, but also to develop a central platform for a wide group of clinicians, scientists and other stakeholders to synergistically work toward a cure for coma.

### Deliverables

The deliverables of the LTR WG include the following:

1. Development of multidisciplinary integrative care model
2. Modeling long-term trajectories across the life span
3. Incorporating technology to optimize LTR
4. Incorporating biomarkers to assess LTR

To achieve this, the WG proposed a phased approach starting with measures to first, (1) help Define DoC cohort; (2) define data requirement for LTR research; and (3) establish and implement care standards. The second phase would focus on (1) strategies to establish methods to access data in real-time from multiple sources and (2) determine the key data points that can best inform care. In the third phase, the WG suggested implementing clinical cohort studies involving multisite DoC network.

## Ethical Considerations for the Patient in Coma

In response to the large number of ethical considerations raised at the first CCC symposium in September 2020, the Campaign convened the Ethics Module Group. The pillars of biomedical ethics consist of representation of patients who are unable to represent themselves by (1) respecting the patient’s autonomy, (2) using beneficence in all aspects of care, (3) providing nonmaleficent care, and (4) considering justice in all decisions. Relatedly, the Belmont Report recommends all research to include respect for persons, beneficence, and justice. The primary ethical concern for patients in coma is their loss of capacity related to decision-making including matters of clinical care and research. As a community involved in the care of the vulnerable population, we all need to work toward identifying and addressing existing ethical conflicts related to patients with DoC as well as proactively anticipate and mitigate potential future ethical conflicts central to the provision of care and the advancement of research [101]. To achieve ethical competence, we need following considerations:

- Care of the comatose patient:
- Accurate diagnosis and differentiation between states of consciousness which has implications for prognostication including discharge decisions.
- Age-based considerations.
- Allocation of resources with respect to management of patients with DoC with consideration of patients without DoC.
- Continuity along the spectrum of care from hospital admission to rehabilitation and finally home/community.
- Decision-making about withdrawing or withholding life-sustaining therapies.
- Ethics of diagnostic modalities in terms of availability and allocation.
- Experimental/translational interventions.
- Practice variation.
- Prognostication.
- Avoid self-fulfilling prophecy or nihilism versus medical futility.
- Family of the comatose patients:
- Family support through the continuum of care.
- Appropriate communication in the ICU.
- Decision-making about goals of care including issues related to advanced care planning (e.g., will) and identification of surrogate decision maker.
- Smooth process of care transitions.
- Burden of care for family.
- Clinical research about coma
- Balance the risks and benefits for patients and family members.
- Informed consent practices.
- Equitable recruitment.
- Standardizing protocols for detection of consciousness.
- Management of data from comatose patients
- Safety, efficacy, and subject selection for investigational drugs and devices.
- Disclosure of results to family and medical team.
- Sharing research results.
- Protection, privacy, and confidentiality of neural data from data mining and discrimination of patients.
- Transparency to families and medical team.
- Data sharing.
- Implementation/innovation for comatose patients
- Distinction between clinical care and research.
- Distinction between clinical and research data.
- Determination of when data can and should be used for actionable diagnosis or prognosis.
- Feasibility of various clinical research technologies.
- Translating research into practice.
- Equity for comatose patients
- Allocation of detection of consciousness, hospital, rehabilitation, and homecare resources.
- Additional considerations such as cultural, international, religious, and social.
- Public engagement and perceptions
- Encourage public education.
- Overcome historical biases and nihilism in the broad medical community and public.
- Share results with the broad medical community, media, and the public.

As a global research platform, the CCC community should adopt above ethical considerations as guiding principles for DoC research.

## Executive Summary

The CCC has achieved great progress since its announcement in October 2019 at the 17th NCS annual meeting. Many field leaders and NCS members across various workgroups and models are actively involved in propelling this colossal mission while remaining inclusive (moon shot culture), adaptable (learn as we go) and available (global platform for coma science). The highly engaging discussions over the 3-day NINDS-funded symposium have elucidated the high degree of overlap across the research spectrum that can only be accomplished with collaborative and synergistic workflow.

The main discussions that took place during the 3-day symposium can be synthesized into three main points: (1) major barriers that have prevented a cure for coma; (2) existing needs to find a cure for coma; and (3) main priorities of the CCC to move its mission forward.

(I) **Major barriers that have prevented a cure for coma**
• Absence of clearly defined problems.
• Inability to accurately identify the type and locations of the defects.
• Unavailability of reliable diagnostic tools.
• Division into separate disease categories (e.g., stroke, TBI).
• Pervasiveness of silo mentality some of which is dependent on how the work force is incentivized.
• Exclusive rather than all-inclusive teams.
• Shortage/Lack of appropriate funding.

(II) **Existing needs to find a cure for coma**

The main research needs for curing coma can be broadly categorized into three main components:

- Infrastructure: Includes database, clinical trial standards, CDEs and strategic collaboration.
- Basic research for cure: Includes understanding of the biology of coma, development of biomarkers for diagnosis and prognostication, preclinical model/animal studies and prospective studies.
- Care of the coma patient: This includes understanding of effectiveness and timing of current routine tests, treatments and interventions, evidence-based approach to reduce practice variability, research related to prognostication and LTR.

Other needs to ensure the success of this mission include:

- An insight into what has prevented a cure for coma.
- Determination of the scope of research needed.
- Understanding of the currently available tools and capabilities.
- Identification of new tools and technologies needed to cure coma.
- Estimation of resources (e.g., funding, database) required to cure coma.
- Elimination of major barriers that hinder our ability to cure coma.
- Risk assessment as well as the development of mitigation strategies.
- Development of action plan with milestones.

(III) **Main priorities of the CCC**
A. Improve patient care based on what we know and can do today to raise the standard of care and improve outcomes. This involves:
  1. Capitalize on existing data sets.
  2. Prospective multicenter interdisciplinary studies to define the science of caring for patients with DoC that creates the optimal context in which recovery can occur.
  3. Develop CDEs/common language.
  4. Analysis and communication of best practices.
B. Understand coma at the neuronal level and defining endotypes to understand the nature and location of the defect and its potential outcome trajectory. For this we need to:
  1. Leverage existing biomarkers and combine them in novel ways.
  2. Develop new biomarkers.
  3. Progress from clinical phenotypes to endotypes for evaluation of dynamic pathobiological changes over time.
  4. Validate with large-scale, multicenter studies.
C. Develop individualized targeted treatments based on endotypes to repair or retrain brain circuits. This can be achieved by means of various approaches, including:
  1. Electromagnetic
  2. Mechanical
  3. Sensory
  4. Chemical
  5. Regenerative
D. Establish prognostication tools with ethical considerations to eliminate nihilism, reduce errors in WOLST and develop objective and empathetic communication strategies with families and surrogates. For this we need:
  1. New tools and models for prognostication to better define the factors that predict outcomes.
  2. Ability to detect cognitive motor dissociation.
  3. Decision support for WOLST.
  4. Establishment of programs of excellence (e.g., CCC faculty scholars’ program where trainees would have the opportunity to learn about best ways of communication with patients and families).
E. Establishment of standards for DoC trials and implementation of these standards to conduct trials aimed at closing current gaps in DoC knowledge. This approach will help foster consistency in data collection and optimize data analysis. Some strategies to reduce research heterogeneity include,
  1. Patients enrollment based on physiologic and anatomic response to targeted therapies
  2. Execution of novel early-phase clinical trials to refine outcome endpoints, and surrogate neuroimaging and biomarkers to improve classification of patients with coma and DoC for future design of larger clinical trials
  3. Development of large-scale, randomized, controlled trials
  4. Utilization of existing tools while focusing on development of new tools, CDEs and biomarkers for standardization.
F. Develop and implement a database that includes clinical, imaging, electrophysiological, chemical, epigenetic and other data. This will create a central repository of relevant DoC data for research collaborations and eventually the development of algorithms for real-time development of decision support tools at the bedside,
G. Insist and ensure that every trial, technical thrust and research effort is transparent, is complementary and builds upon all the other efforts. This approach will help eliminate silos and reduce redundancy and waste.
H. Establish an effective new approach for DoC. This involves the development of new model for central data and management center. One approach could be “research focus specific” where 3–5 centers would collaborate to achieve goals specific to each working group with defined milestones within a 5-year funding cycle. In this model, about 30 centers would participate to propel the CCC mission in parallel while remaining highly engaged and communicative where results of one group would inform the study design of the other group within the CCC collaborative. In this model, the CCC collaborative would undergo quarterly reviews of performance and outcomes and an annual review by an advisory board while ensuring collaboration and lack of redundancy. The collaboration would remain adaptive with the addition of new centers as needed and elimination of underperforming centers. Such a model could allow lifetime follow-up of all enrolled participants across the research domains.

The CCC executive committee has estimated a total budget of US $60 million (about 12 million per year) to accomplish these goals. Estimates related to various priority areas include:

- Database development: 2 million
- Biology and new biomarker development: 15 million
- Prospective studies: 10 million
- Clinical trials for treatment evaluations: 13 million
- Patient care protocol development: 10 million
- Prognostication and ethics: 10 million

Over the past 10 years, about 24.3 million (average of 2.4 million/year) NIH dollars have already been invested in DoC research with a total of 68 awardees, only 6 of which are active awards [102]. Meanwhile, the societal cost of coma has exceeded $75 billion with 25% of the total health care cost related only to ICU stay! Given this magnitude of economic burden related to DoC, even if efforts related to CCC improve care and reduce cost by 10%, the return of investment will be enormous.

## Conclusions

The Curing Coma community has made significant progress since its inception. Now is the time to collate efforts not only to improve the understanding of mechanistic underpinnings of coma, but also to find ways to translate scientific discovery into clinical practice. It is crucial to bridge siloed independent efforts globally with a communicative, collaborative and synergistic strategy from multidisciplinary clinical and research teams across the continuum of coma care. To facilitate this collaborative approach, a centralized platform is urgently needed to bring together clinicians, scientists, patients, and families for a common mission of improving care of the comatose patients. Establishment of such a platform with clear vision and defined milestones is the natural next step. This platform would be critical not only in educating and galvanizing the stakeholders and creating the global standards of coma care but also in improving the outcomes of patients with disordered consciousness.

## Supplementary Information

Below is the link to the electronic supplementary material.

Supplementary file1 (DOCX 44 kb)
