# Correction to: Proceedings of the Second Curing Coma Campaign NIH Symposium: Challenging the Future of Research for Coma and Disorders of Consciousness

**DOI:** 10.1007/s12028-022-01536-w

**Published:** 2022-06-17

**Authors:** Shraddha Mainali, Venkatesh Aiyagari, Sheila Alexander, Yelena Bodien, Varina Boerwinkle, Melanie Boly, Emery Brown, Jeremy Brown, Jan Claassen, Brian L. Edlow, Ericka L. Fink, Joseph J. Fins, Brandon Foreman, Jennifer Frontera, Romergryko G. Geocadin, Joseph Giacino, Emily J. Gilmore, Olivia Gosseries, Flora Hammond, Raimund Helbok, J. Claude Hemphill, Karen Hirsch, Keri Kim, Steven Laureys, Ariane Lewis, Geoffrey Ling, Sarah L. Livesay, Victoria McCredie, Molly McNett, David Menon, Erika Molteni, DaiWai Olson, Kristine O’Phelan, Soojin Park, Len Polizzotto, Jose Javier Provencio, Louis Puybasset, Chethan P. Venkatasubba Rao, Courtney Robertson, Benjamin Rohaut, Michael Rubin, Tarek Sharshar, Lori Shutter, Gisele Sampaio Silva, Wade Smith, Robert D. Stevens, Aurore Thibaut, Paul Vespa, Amy K. Wagner, Wendy C. Ziai, Elizabeth Zink, Jose I. Suarez, the Curing Coma Campaign collaborators

**Affiliations:** 1 Department of Neurology, Virginia Commonwealth University School of Medicine, Richmond, VA USA; 2 Neurological Surgery and Neurology, University of Texas Southwestern Medical Center, Dallas, TX USA; 3 School of Nursing, University of Pittsburgh, Pittsburgh, PA USA; 4 Department of Physical Medicine and Rehabilitation, Spaulding Rehabilitation Hospital and Harvard Medical School, Charlestown, MA USA; 5 Division of Neurology, Barrow Neurological Institute at Phoenix Children’s Hospital, Phoenix, AZ USA; 6 Departments of Neurology and Psychiatry, Wisconsin Institute for Sleep and Consciousness, University of Wisconsin, Madison, WI USA; 7 Department of Anesthesia, Critical Care and Pain Medicine, Massachusetts General Hospital and Harvard Medical School, Boston, MA USA; 8 Office of Emergency Care Research, Division of Clinical Research, National Institute of Neurological Disorders and Stroke, Bethesda, MD USA; 9 Department of Neurology, Columbia University Medical Center, New York Presbyterian Hospital, New York, NY USA; 10 Department of Neurology, Center for Neurotechnology and Neurorecovery, Massachusetts General Hospital, Boston, MA USA; 11 Department of Critical Care Medicine, UPMC Children’s Hospital of Pittsburgh, University of Pittsburgh Medical Center, Pittsburgh, PA USA; 12 Division of Medical Ethics, Weill Cornell Medical College, New York, NY USA; 13 Division of Neurocritical Care, Department of Neurology and Rehabilitation Medicine, University of Cincinnati, Cincinnati, OH USA; 14 Department of Neurology, New York University School of Medicine, New York, NY USA; 15 Division of Neurosciences Critical Care, Departments of Anesthesiology and Critical Care Medicine, Neurology, and Neurosurgery, The Johns Hopkins University School of Medicine, Baltimore, MD USA; 16 Harvard Medical School, Spaulding Rehabilitation Hospital, Boston, MA USA; 17 Comprehensive Epilepsy Center, Department of Neurology, Yale University, New Haven, CT USA; 18 Coma Science Group, GIGA Consciousness, University of Liege, Liege, Belgium; 19 Indiana University Department of Physical Medicine and Rehabilitation, University of Indiana School of Medicine, Indianapolis, IN USA; 20 Department of Neurology, Medical University of Innsbruck, Innsbruck, Austria; 21 Department of Neurology, University of California, San Francisco, CA USA; 22 Division of Neurocritical Care, Department of Neurology, Stanford University, Stanford, CA USA; 23 College of Pharmacy, University of Illinois, Chicago, IL USA; 24 Coma Science Group, Cyclotron Research Center, University of Liege, Liege, Belgium; 25 Department of Neurology and Neurosurgery, New York University Langone Health, New York, NY USA; 26 Department of Adult Health and Gerontological Nursing, College of Nursing, Rush University, Chicago, IL USA; 27 Interdepartmental Division of Critical Care, Department of Respirology, University of Toronto, Toronto, ON Canada; 28 College of Nursing, Ohio State University, Columbus, OH USA; 29 Division of Anaesthesia, University of Cambridge, Cambridge, UK; 30 School of Biomedical Engineering and Imaging Sciences, King’s College London, London, UK; 31 Neuroscience Intensive Care Unit, O’Donnell Brain Institute, University of Texas Southwestern Medical Center, Dallas, TX USA; 32 Department of Neurology, Miller School of Medicine, University of Miami, Miami, FL USA; 33 Department of Neurology and Neurocritical Care, Columbia University, New York, NY USA; 34 Department of Biomedical Engineering, Worcester Polytechnic Institute, Worcester, MA USA; 35 Department of Neurology and Neuroscience, University of Virginia, Charlottesville, VA USA; 36 Department of Neuroradiology, University of Paris VI, Pierre et Marie Curie, Pitié-Salpêtrière Hospital, Paris, France; 37 Division of Vascular Neurology and Neurocritical Care, CHI St. Luke’s Health–Baylor St. Luke’s Medical Center, Baylor College of Medicine, Houston, TX USA; 38 Departments of Anesthesiology and Critical Care Medicine, and Pediatrics, Johns Hopkins Children’s Center, The Johns Hopkins University School of Medcine, Baltimore, MD USA; 39 Neuroscience Intensive Care Unit, Department of Neurology, Sorbonne University, Assistance Publique–Hôpitaux de Paris, Pitié-Salpêtrière Hospital, Paris, France; 40 Department of Intensive Care, Paris Descartes University, Paris, France; 41 University of Pittsburgh, Pittsburgh, PA USA; 42 Hospital Israelita Albert Einstein, Academic Research Organization and Department of Neurology and Neurosurgery, Universidade Federal de São Paulo, São Paulo, Brazil; 43 Ronald Reagan UCLA Medical Center, UCLA Santa Monica Medical Center, Santa Monica, CA USA; 44 Department of Physical Medicine and Rehabilitation, School of Medicine, University of Pittsburgh, Pittsburgh, PA USA; 45 Department of Neuroscience Nursing, The Johns Hopkins Hospital, The Johns Hopkins University, Baltimore, MD USA; 46 Athinoula A. Martinos Center for Biomedical Imaging, Massachusetts General Hospital, Charlestown, MA USA; 47 Yale Law School, New Haven, CT USA; 48 Centre du Cerveau, University Hospital of Liege, Liege, Belgium; 49 Department of Neurology, Centre Hospitalier Universitaire Sart Tilman, University of Liege, Liege, Belgium

## Correction to: Neurocrit Care 10.1007/s12028-022-01505-3

Article has been updated to correct Dr. Ericka L. Fink’s name.

