# Supplementary material for: Proceedings of the Second Curing Coma Campaign NIH Symposium: Challenging the Future of Research for Coma and Disorders of Consciousness
Source: Neurocrit Care. 2022 May 10;37(1):326–50. doi: 10.1007/s12028-022-01505-3 (PMC9283342; doi:10.1007/s12028-022-01505-3)
Supplement: Supplementary file 1 — Supplementary file1 (DOCX 44 kb) [file 12028_2022_1505_MOESM1_ESM.docx]

# Appendix 1: Meeting Agenda

**DAY 1 – MONDAY, MAY 3, 2021**

| **Time (EDT)** | **Session Title** |
| --- | --- |
| 10:00 AM – 10:05 AM | Opening Remarks  *Jeremy Brown, Claude Hemphill, Walter Koroshetz, DaiWai Olson and Jose I Suarez* |
| 10:05 AM – 10:25 AM | Curing Coma NIH Symposium Part I  *Jan Claassen* |
| 10:25 AM – 10:45 AM | Defining Coma and Disorders of Consciousness  *Benjamin Rohaut* |
| 10:45 AM – 11:05 AM | Stakeholder: The Patient’s Perspective  *Anezi Uzendu* |
| 11:05 AM – 11:25 AM | Keynote Presentation: Why Are We Here?  *Geoffrey Ling* |
| 11:25 AM – 11:35 AM | Break |
| 11:35 AM – 11:55 | The Biology of Consciousness  *David Menon* |
| 11:55 AM – 12:25 PM | Session 1: Research Priorities for Biology of Coma; Panel Discussion; Session Summary  *Session Co-Chairs: Sheila Alexander and Melanie Boly Speaker: Olivia Gosseries* |
| 12:25 PM – 12:55 PM | Session 1 Panel Discussion  *Panelists: Olivia Gosseries, Courtney Robertson, Robert Stevens, and Aurore Thibaut* |
| 12:55 PM – 1:00 PM | Session 1 Summary/Comments  *Summary Presenter: Javier Provencio* |
| 1:00 PM – 1:10 PM | Break |
| 1:10 PM – 1:30 PM | Common Data Elements in Coma Research  *Jan Claassen* |
| 1:30 PM – 2:00 PM | Session 2: Research Priorities for Coma Database; Panel Discussion; Session Summary  *Session Co-Chairs: Brandon Foreman and Emily Gilmore* |
|  | *Speaker: Paul Vespa* |
| 2:00 PM – 2:30 PM | Session 2 Panel Discussion  *Panelists: Louis Puybasset, Chethan Venkatasubba Rao, and Paul Vespa* |
| 2:30 PM – 2:35 PM | Session 2 Summary/Comments  *Summary Presenter: Soojin Park* |
| 2:35 PM – 2:45 PM | Closing Remarks |

**DAY 2 – TUESDAY, MAY 4, 2021**

| **Time (EDT)** | **Session Title** |  |
| --- | --- | --- |
| 10:00 AM – 10:20 AM | Saul Bellow’s Coma: What Neuroscience Can Learn From Humanities  *Joseph Fins* |  |
| 10:20 AM – 10:50 AM | Session 3: Research Priorities for Prognostication  *Session Co-Chairs: Romergryko Geocadin and Steven Laureys Speaker: Wendy Ziai* |  |
| 10:50 AM – 11:20 AM | Session 3 Panel Discussion  *Panelists: Erika Fink, Michael Rubin, and Wendy Ziai* |  |
| 11:20 AM – 11:25 AM | Session 3 Summary/Comments  *Summary Presenter: Karen Hirsch* |  |
| 11:25 AM – 11:35 AM | Break |  |
| 11:35 AM – 11:55 AM | Acute Care of the Patient During Coma  *DaiWai Olson* |  |
| 11:55 AM – 12:25 AM | Session 4: Research Priorities for Care of the Comatose and Disorder of Consciousness Patient  *Session Co-Chairs: Keri Kim and Elizabeth Zink Speaker: Molly McNett* |  |
| 12:25 PM – 12:55 PM | Session 4 Panel Discussion  *Panelists: Varina Boerwinkle, Sarah Livesay, Molly McNett, and Gisele Silva* |  |
| 12:55 PM – 1:00 PM | Session 4 Summary/Comments  *Summary Presenter: Venkatesh Aiyagari* |  |
| 1:00 PM – 1:10 PM | Break |  |
| 1:10 PM – 1:30 PM | Anesthesia and Consciousness  *Emery Brown* | |
| 1:30 PM – 2:00 PM | Session 5: Research Priorities for Early Clinical Trials *Session Co-Chairs: Joseph Giacino and Wade Smith Speaker: Lori Shutter* | |
| 2:00 PM – 2:30 PM | Session 5 Panel Discussion  *Panelists: Yelena Bodien, Brian Edlow, Raimund Helbok, and Lori Shutter* | |
| 2:30 PM – 2:35 PM | Session 5 Summary/Comments  *Summary Presenter: Victoria McCredie* | |
| 2:35 PM – 2:45 PM | Break | |
| 2:45 PM – 3:15 PM | Session 6: Research Priorities for Long-term Recovery *Session Co-Chairs: Jennifer Frontera and Tarek Sharshar Speaker: Amy Wagner* | |
| 3:15 PM – 3:45 PM | Session 6 Panel Discussion  *Panelists: Erika Molteni, Kristine O’Phelan, Michelle Schober, and Amy Wagner* | |
| 3:45 PM – 3:50 PM | Session 6 Summary/Comments  *Summary Presenter: Flora Hammond* | |
| 3:50 PM – 4:10 PM | Ethical Considerations for the Patient in Coma  *Ariane Lewis* | |
| 4:10 PM – 4:20 PM | Closing Remarks  *Jeremy Brown, Claude Hemphill, DaiWai Olson, and Jose I Suarez* | |

**DAY 3 – WEDNESDAY, MAY 5, 2021**

| **Time (EDT)** | **Session Title** |  |
| --- | --- | --- |
| 10:00 AM – 10:15 AM | Introduction and Overview  *Len Polizzotto* |  |
| 10:15 AM – 10:45 AM | Group 1: Biology of Coma – Priorities  *Olivia Gosseries and Javier Provencio* |  |
| 10:45 AM – 11:15 AM | Group 2: Coma Database – Priorities  *Soojin Park and Paul Vespa* |  |
| 11:15 AM – 11:25 AM | Break |  |
| 11:25 AM – 11:55 AM | Group 3: Prognostication – Priorities  *Karen Hirsch and Wendy Ziai* | |
| 11:55 AM – 12:25 PM | Group 4: Care of the Comatose Patient – Priorities  *Venkatesh Aiyagari and Molly McNett* | |
| 12:25 PM – 12:30 PM | Break | |
| 12:30 PM – 1:00 PM | Group 5: Early Clinical Trials – Priorities  *Victoria McCredie and Lori Shutter* | |
| 1:00 PM – 1:30 PM | Group 6: Long-term Recovery – Priorities  *Flora Hammond and Amy Wagner* | |
| 1:30 PM – 1:40 PM | Break | |
| 1:40 PM – 2:30 PM^[[1]](#endnote-1)^ | Summary and Final Recommendations  *Len Polizzotto* | |

1. **APPENDIX 2**

   **List of attendees NINDS Curing Coma® Symposium May 3-5, 2021**

   | Sachin | Agarwal | sa2512@columbia.edu |
   | --- | --- | --- |
   | Shefali | Aggarwal | saggarwal@phoenixchildrens.com |
   | Venkatesh | Aiyagari | venkatesh.aiyagari@utsouthwestern.edu |
   | Ayham | Alkhachroum | axa2610@med.Miami.edu |
   | Sharon | Allen | sallen@neurocriticalcare.org |
   | Rashid | Alaraimi | rashid.alaraimi@mail.mcgill.ca |
   | Sheila | Alexander | salexand@pitt.edu |
   | Ayham | Alkhachroum | axa2610@med.Miami.edu |
   | Sharon | Allen | sallen@neurocriticalcare.org |
   | Mary | Amatangelo | Mamatangelo@bwh.Harvard.edu |
   | Pouya | Ameli | pouya@ufl.edu |
   | Edilberto | Amorim | amorim@ucsf.edu |
   | Beth | Ansel | beansel@iu.edu |
   | Neeraj | Badjatia | nbadjatia@som.umaryland.edu |
   | James | Bartscher | jfbartscher@gmail.com |
   | Rachel | Beekman | Rachel.beekman@yale.edu |
   | Andrey | Belkin | belkin@neuro-ural.ru |
   | Vladimir | Belkin | vbelkin@neuro-ural.ru |
   | Patrick | Bellgowan | psfb@mail.nih.gov |
   | Victor | Bello | vubb_2909_umq@hotmail.com |
   | Rebecca | Berman | bermanr@mail.nih.gov |
   | Shubhayu | Bhattacharyay | sb2406@cam.ac.uk |
   | Marek | Binder | marek.binder@uj.edu.pl |
   | Stefanie | Blain-Moraes | stefanie.blain-moraes@mcgill.ca |
   | Karolyna | Blanco | Karolax@hotmail.com |
   | Thomas | Bleck | tbleck@gmail.com |
   | Yelena | Bodien | ybodien@mgh.harvard.edu |
   | Varina | Boerwinkle | vboerwinkle@phoenixchildrens.com |
   | Melanie | Boly | boly@neurology.wisc.edu |
   | Estelle | Bonin | estelle.bonin@uliege.be |
   | Mario | Bouly Castro | boulymario22@gmail.com |
   | Marie-Michele | Briand | marie-michele.briand.1@ulaval.ca |
   | Emery | Brown | enb@neurostat.mit.edu |
   | Jeremy | Brown | jeremy.brown@nih.gov |
   | Martina | Cacciatore | marty9484@live.it |
   | eusebia | calvillo | ecalvil2@jhmi.edu |
   | Elizabeth | Carroll | Elizabeth.carroll@nyumc.org |
   | marilena | casartelli liviero | marilena.casartelliliviero@aovr.veneto.it |
   | Meghan | Caylor | meghan.caylor@pennmedicine.upenn.edu |
   | Cherylee W | Chang | cherylee.chang@duke.edu |
   | Lily | Chau | chau.lily@gmail.com |
   | Sung-Min | Cho | csmfisher@gmail.com |
   | Richard | Choi | richardkchoi@gmail.com |
   | Lily | Chau | chau.lily@gmail.com |
   | Melissa | Chung | melissa.chung@nationwidechildrens.org |
   | Jan | Claassen | jc1439@columbia.edu |
   | Erin | Cole | erin.cole@mcgill.ca |
   | Elizabeth | Crago | ecrago@pitt.edu |
   | Patrick | Cullinan | PatrickCullinanDO@gmail.com |
   | Francesco | Curto | francesco.curto@ospedaleniguarda.it |
   | LUIZ | DALFIOR JUNIOR | dalfiorjunior@gmail.com |
   | Elizabeth | Diaz | diaze@musc.edu |
   | Camilo | Diaz Cruz | cdiazcr1@jhmi.edu |
   | Michele | Dillon | michele.dillon@roseliassociates.com |
   | Michael | Diringer | diringerm@wustl.edu |
   | Kevin | Doyle | kd2630@cumc.columbia.edu |
   | Catherine | Duclos | catherine.duclos@mail.mcgill.ca |
   | Peter | Dziedzic | phd@jhu.edu |
   | Brian | Edlow | bedlow@mgh.harvard.edu |
   | Ahmed | Elkady | ahkady86@gmail.com |
   | Leon | Epstein | l-epstein@northwestern.edu |
   | Salia | Farrokh | salia.farrokh@gmail.com |
   | Corey | Fehnel | cfehnel@bidmc.harvard.edu |
   | Elana | Felder | efelder@binausa.org |
   | Simona | Ferioli | simonaferioli@gmail.com |
   | MONICA | FERREA | mferrea@gmail.com |
   | Ericka | Fink | finkel@ccm.upmc.edu |
   | Joseph | Fins | jjfins@med.cornell.edu |
   | Jennifer | Fisher | jlfisher78@gmail.com |
   | Brandon | Foreman | foremabo@ucmail.uc.edu |
   | Conall | Francoeur | conall.francoeur.med@ssss.gouv.qc.ca |
   | Allison | Frantz | allison.frantz@mail.mcgill.ca |
   | Herbert | Fried | brainzrus@mac.com |
   | Jennifer | Frontera | Jennifer.Frontera@nyulangone.org |
   | Richard | Frye | rfrye@phoenixchildrens.com |
   | Marie-Ève | Gagnon | mgagno42@uwo.ca |
   | Romer | Geocadin | rgeocad1@jhmi.edu |
   | maryam | ghaleh | maryam.ghaleh@nih.gov |
   | Joseph | Giacino | jgiacino@mgh.harvard.edu |
   | Christie | Gibbons | cbutlergibbons@gmail.com |
   | Emily | Gilmore | emily.gilmore@yale.edu |
   | Chavie | Glustein | cglustein@binausa.org |
   | Florent | GOBERT | gobert.flo@gmail.com |
   | FERNANDO | GOLDENBERG | fgoldenb@neurology.bsd.uchicago.edu |
   | Urszula | Górska | gorska@wisc.edu |
   | Olivia | Gosseries | ogosseries@uliege.be |
   | ELENA | GRAPPA | elena.grappa@asst-cremona.it |
   | Shelby | Halsey | shelby.halsey@utsouthwestern.edu |
   | Flora | Hammond | flora.Hammond@rhin.com |
   | Amy | Hamrick | amyhamrick@srhs.com |
   | Adam | Hartman | adam.hartman@nih.gov |
   | Raimund | Helbok | raimund.helbok@tirol-kliniken.at |
   | Claude | Hemphill | claude.hemphill@ucsf.edu |
   | Archana | Hinduja | Archana.hinduja@osumc.edu |
   | H E | Hinson | hinson@ohsu.edu |
   | Karen | Hirsch | khirsch@stanford.edu |
   | Rebecca | Hommer | rebecca.hommer@nih.gov |
   | Xiao | Hu | xiao.hu@duke.edu |
   | Theresa | Human | theresa.human@bjc.org |
   | Ajit | Indavarapu | Ajit.2548aj@gmail.com |
   | Isamu | Isozaki | isamu.website@gmail.com |
   | Firas | Kaddouh | firaskaddouh@gmail.com |
   | Anna | Karpenko | anna.karpenko@hitchcock.org |
   | Kasia | Kasica | Stemcells@akogo.pl |
   | Douglas | Katz | dkatz@bu.edu |
   | Karnig | Kazazian | kkazazia@uwo.ca |
   | David E | Kahn | d.ethan.kahn@nyulangone.org |
   | Imad | Khan | imad_khan@urmc.rochester.edu |
   | Keri | Kim | skim42@uic.edu |
   | Matthew | Kirschen | kirschenm@chop.edu |
   | Hitoshi | Kobata | neu035@osaka-med.ac.jp |
   | Jim | Koenig | jim.koenig@nih.gov |
   | Ekaterina | Kondratyeva | eak2003@mail.ru |
   | Daniel | Kondziella | daniel_kondziella@yahoo.com |
   | Walter | Koroshetz | koroshetzw@ninds.nih.gov |
   | Lyudmila | Korostovtseva | Lyudmila_korosto@mail.ru |
   | Csaba | Kozma | csaba.a.kozma@gmail.com |
   | Pedro | Kurtz | kurtzpedro@mac.com |
   | Kerri | LaRovere | kerri.larovere@childrens.harvard.edu |
   | Steven | Lawrence | lawrencemsteven@gmail.com |
   | Steven | Laureys | steven.laureys@ulg.ac.be |
   | Thomas | Lawson | thomas.lawson@osumc.edu |
   | Tyler | Le | tml95@drexel.edu |
   | matilde | leonardi | matilde.leonardi@istituto-besta.it |
   | Ariane | Lewis | ariane.kansas.lewis@gmail.com |
   | Karen | Lidsky MD | karen.lidsky@jax.ufl.edu |
   | Geoff | Ling | gling1@jhmi.edu |
   | Sarah | Livesay | Sarah_L_Livesay@rush.edu |
   | Marlina | Lovett | marlina.lovett@nationwidechildrens.org |
   | Bethany | Lussier | Bethany.Lussier@utsouthwestern.edu |
   | Heather | Ma | Heather_Ma@urmc.rochester.edu |
   | Lori | Madden | lkmadden@ucdavis.edu |
   | Craig | Maddux | craig.maddux@us.ibm.com |
   | Shraddha | Mainali | shraddha.mainali@vcuhealth.org |
   | Allie | Mandel | amandel@neurocriticalcare.org |
   | Tara | Mangum | tmangum@phoenixchildrens.com |
   | Iris | Marku | imarku@phoenixchildrens.com |
   | Sofia | Martinez | sofias.mtzr@gmail.com |
   | Beril | Mat | mat@neurology.wisc.edu |
   | Stephan | Mayer | Stephanamayer@gmail.com |
   | Victoria | McCredie | Victoria.McCredie@uhn.ca |
   | Beth | McGuirk | bmcguirk@phoenixchildrens.com |
   | Molly | McNett | mcnett.21@osu.edu |
   | Murad | Megjhani | megjhani@gmail.com |
   | Jorge | Mejia-Mantilla | jorge.mejia.m@me.com |
   | Carolina | Mendoza-Puccini | Carolina.Mendoza-Puccini@nih.gov |
   | David | Menon | dkm13@cam.ac.uk |
   | Benjamin | Miller | brmille@umn.edu |
   | Dick | Moberg | dick@moberganalytics.com |
   | Erika | Molteni | erika.molteni@kcl.ac.uk |
   | elena | monai | elena24mn@gmail.com |
   | Martin | Monti | monti@mednet.ucla.edu |
   | Sulimar | Morales | Sulimar.morales@gmail.com |
   | Melissa | Motta | mmotta@som.umaryland.edu |
   | Ethan | Moyer | ejm374@drexel.edu |
   | Susanne | Muehlschlegel | susanne.muehlschlegel@umassmemorial.org |
   | Michele | Munkwitz | mmunkwitz@phoenixchildrens.com |
   | Brooke | Murtaugh | bmurtaugh@madonna.org |
   | JAGARLAPUDI M | MURTHY | jmkmurthy49@gmail.com |
   | Masao | Nagayama | nagay001@iuhw.ac.jp |
   | Julia | Nekrasova | nekrasova84@yandex.ru |
   | Masachika | Niimi | pomardon2010@gmail.com |
   | Filipa | Noronha Falcão | fnoronhafalcao@gmail.com |
   | Paul | Nyquist | pnyquis1@jhmi.edu |
   | Paul | Nyquist | pnyquis1@jhmi.edu |
   | Nicole | OBrien | nicole.obrien@nationwidechildrens.org |
   | Katherine | O'Brien | katherine.obrien@memorialhermann.org |
   | John | O'Donnell | john.charles.odonnell@gmail.com |
   | DaiWai | Olson | daiwai.olson@utsouthwestern.edu |
   | Kristine | O'Phelan | kophelan@med.miami.edu |
   | Carlos | Orellana Jimenez | ucicealojim@yahoo.com |
   | Soojin | Park | spark@columbia.edu |
   | Shash | Patel | spatel11@phoenixchildrens.com |
   | Katrina | Peariso | peariska@ucmail.uc.edu |
   | Anton | Peled | antonpeled@gmail.com |
   | Patrick | Pittermann | patrick.pittermann@web.de |
   | Len | Polizzotto | len_polizzotto@yahoo.com |
   | Douglas | Postels | dpostels@childrensnational.org |
   | Javier | Provencio | jp3b@virginia.edu |
   | Vernita | Pupuma | vpupuma@iqsolutions.com |
   | Louis | Puybasset | louis.puybasset@psl.aphp.fr |
   | Abid | Qureshi | abid.y.qureshi@gmail.com |
   | Shamsi | Raeissi | Raeissi09@gmail.com |
   | Chethan Venkatasubba | Rao | cprao@bcm.edu |
   | Lindsey | Rasmussen | lkrasmus@stanford.edu |
   | Bappaditya | Ray | Bappaditya.Ray@UTSouthwestern.edu |
   | partha | ray | partha_ray@hotmail.com |
   | Chris | Reeves | chrisreeves7@gmail.com |
   | Mohammed | Rehman | mrehman1@hfhs.org |
   | Ranier | Reyes | ranier.reyes@utsouthwestern.edu |
   | Risa | Richardson | Risa.Richardson@va.gov |
   | LUCIA | RIVERA LARA | lucyriveral@gmail.com |
   | Courtney | Robertson | crober48@jhmi.edu |
   | Toya | Rogers | toya.rogers@nih.gov |
   | Benjamin | Rohaut | benjamin.rohaut@aphp.fr |
   | David Pascual | Rojas Flores | pediatriacompleta@gmail.com |
   | Amy | Rosenbaum | Ashapi1@hotmail.com |
   | Kate | Rosenblatt | krosenb3@jhmi.edu |
   | Andres | Rubiano | rubianoam@gmail.com |
   | Daniel | Rubin | drubin4@mgh.harvard.edu |
   | Michael | Rubin | michael.rubin@utsouthwestern.edu |
   | Maria Julieta | Russo | jrusso@fleni.org.ar |
   | ALOK | SAHGAL | alok.sahgal@gmail.com |
   | Leandro | Sanz | leandro.sanz@uliege.be |
   | davide | sattin | davide.sattin@istituto-besta.it |
   | Caroline | Schnakers | cschnakers@casacolina.org |
   | Michelle | Schober | michelle.schober@hsc.utah.edu |
   | Julie | Schwertfeger | Julie.Schwertfeger@rosalindfranklin.edu |
   | steven | scott | steven.scott@va.gov |
   | Ekta | Shah | ekta.g.shah@uth.tmc.edu |
   | Tarek | Sharshar | tsharshar@gmail.com |
   | Qi | Shen | qs2230@cumc.columbia.edu |
   | Lori | Shutter | shutterla@upmc.edu |
   | Robert | Silbergleit | robert.silbergleit@umich.edu |
   | Gisele | Silva | giselesampaio@hotmail.com |
   | Gordon | Siu | gordon.siu@midmichigan.org |
   | Sanjeev | Sivakumar | sanjeev.sivakumar@prismahealth.org |
   | Wade | Smith | smithw@neurology.ucsf.edu |
   | Federico | Sosa Albacete | fsosa@fleni.org.ar |
   | Norman | Spivak | nspivak@mednet.ucla.edu |
   | Joyce | Steenberghs | joyce.steenberghs@jessazh.be |
   | Robert | Stevens | rstevens@jhmi.edu |
   | Jose | Suarez | jsuarez5@jhmi.edu |
   | Lauren | Sutton | lauren.sutton@bjc.org |
   | Jacek | Szczygielski | jacek.szczygielski@vp.pl |
   | Emilie | Szymkowicz | emilie.szymkowicz@uliege.be |
   | Terrie | Taylor | taylort@msu.edu |
   | Aurore | Thibaut | athibaut@uliege.be |
   | Ajay | Thomas | ajay.thomas@bcm.edu |
   | Anezi | Uzendu | uzendu14@gmail.com |
   | Glenn | van der Lande | glenn.vanderlande@uliege.be |
   | Panayiotis | Varelas | varelap@amc.edu |
   | Paul | Vespa | pvespa@mednet.ucla.edu |
   | Walter | Videtta | wvidetta2@icloud.com |
   | Marie | Vitello | marie.vitello@outlook.com |
   | Amy | Wagner | wagnerak@upmc.edu |
   | Mark | Wainwright | mwa110@uw.edu |
   | John | Whyte | jwhyte@einstein.edu |
   | Craig | Williamson | craigaw@med.umich.edu |
   | Frederick | Willyerd | awillyerd@phoenixchildrens.com |
   | Clinton | Wright | clinton.wright@nih.gov |
   | Mais | Yacoub | dr.yacoubmais@gmail.com |
   | Michael | Young | Michael.Young@mgh.harvard.edu |
   | Wendy | Ziai | weziai@jhmi.edu |
   | Elizabeth | Zink | ezink1@jhmi.edu |

   [↑](#endnote-ref-1)
